# Dynamic patterns of microRNA expression during acute myeloid leukemia state-transition

**DOI:** 10.1126/sciadv.abj1664

**Published:** 2022-04-22

**Authors:** David E. Frankhouser, Denis O’Meally, Sergio Branciamore, Lisa Uechi, Lianjun Zhang, Ying-Chieh Chen, Man Li, Hanjun Qin, Xiwei Wu, Nadia Carlesso, Guido Marcucci, Russell C. Rockne, Ya-Huei Kuo

**Affiliations:** 1 Department of Population Sciences, City of Hope National Medical Center, Duarte, CA 91010, USA.; 2 Division of Mathematical Oncology, Department of Computational and Quantitative Medicine, City of Hope National Medical Center, Duarte, CA 91010, USA.; 3 Center for Gene Therapy, City of Hope National Medical Center, Duarte, CA 91010, USA.; 4 Department of Diabetes Complications and Metabolism, City of Hope National Medical Center, Duarte, CA 91010, USA.; 5 Department of Hematological Malignancies Translational Science, Hematology and Hematopoietic Cell Transplantation, City of Hope National Medical Center, Duarte, CA 91010, USA.; 6 The Gehr Family Center for Leukemia Research, City of Hope National Medical Center, Duarte, CA 91010, USA.; 7 Department of Computational and Quantitative Medicine, Integrative Genomics Core, City of Hope National Medical Center, Duarte, CA 91010, USA.; 8 Department of Stem Cell and Regenerative Medicine, City of Hope National Medical Center, Duarte, CA 91010, USA.

## Abstract

MicroRNAs (miRNAs) have been shown to hold prognostic value in acute myeloid leukemia (AML); however, the temporal dynamics of miRNA expression in AML are poorly understood. Using serial samples from a mouse model of AML to generate time-series miRNA sequencing data, we are the first to show that the miRNA transcriptome undergoes state-transition during AML initiation and progression. We modeled AML state-transition as a particle undergoing Brownian motion in a quasi-potential and validated the AML state-space and state-transition model to accurately predict time to AML in an independent cohort of mice. The critical points of the model provided a framework to align samples from mice that developed AML at different rates. Our mathematical approach allowed discovery of dynamic processes involved during AML development and, if translated to humans, has the potential to predict an individual’s disease trajectory.

## Teaser

The microRNA transcriptome undergoes a state-transition during acute myeloid leukemia development and predicts time to disease.

## INTRODUCTION

Acute myeloid leukemia (AML) is a molecularly heterogeneous neoplastic disease originating in the bone marrow (BM) with more than 20,000 new cases diagnosed in the United States each year (*1*). The relatively low 5-year survival rate of 28% reflects the urgent need for more effective treatments. With the rapid advancement and pervasive use of sequencing technologies in the clinical management of AML, there is an opportunity to take advantage of time-sequential multiomic samplings of relevant tissues (BM and peripheral blood), to identify previously unknown targets, and to devise novel therapeutic approaches.

State-transition models have been useful to interpret and predict time-sequential dynamics in stochastic systems. The biological applications of state-transition models include development, cell differentiation, and disease. In the context of diseases, including cancer, state transitions are useful for studying disease initiation and progression. Constructing a state-space to model biological transitions can capture changes produced by a vast number of processes that occur simultaneously in a biological system (*2*, *3*). State-spaces have been constructed using a variety of different types of data, but the mRNA transcriptome is often used because it is relatively easy to assay and contains sufficient information to represent cell states. However, the large amount of information and high dimensionality of the transcriptome also present challenges.

MicroRNAs (miRNAs) are small noncoding RNA molecules involved in posttranscriptional regulation of gene expression. miRNA expression profiles have been associated with pathogenesis and prognosis of AML, yet very little is known about the dynamics of miRNA expression (i.e., changes in miRNA expression over time) during the course of AML initiation and progression or how these dynamics can be targeted therapeutically. To our knowledge, state-transition modeling of the miRNA transcriptome in AML has not been previously reported.

Here, using peripheral blood mononuclear cells (PBMCs) collected at sequential time points from a murine model of inv(16) AML, we show that the miRNA transcriptome undergoes a state-transition from disease initiation to progression. We defined a miRNA state- space and identified state-transition critical points that represent transcriptional states during AML progression. Critical point–based analysis identified miRNA-based regulatory events that predict the dynamics of AML development from a state of perturbed hematopoiesis to overt disease. This approach allowed us also to discover dynamic patterns of miRNA expression in AML development.

## RESULTS

### Construction of an AML state-transition state-space using time-sequential miRNA data

**Fig. 1.**
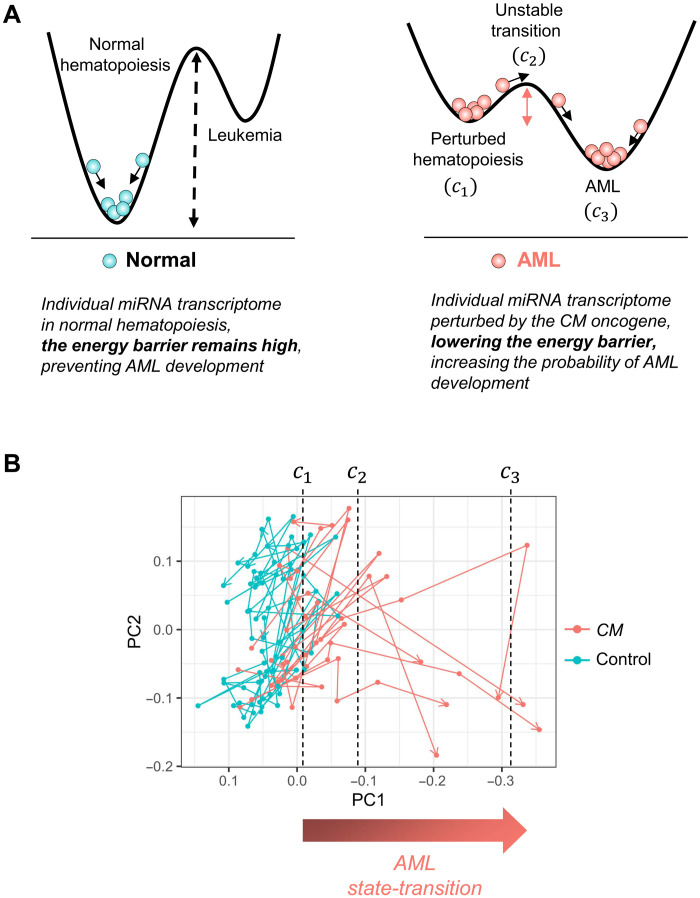
AML state-transition model and state-space. (**A**) Our state-transition model represents the miRNA transcriptome as a particle undergoing Brownian motion in a double-well quasi-potential. The time evolution of the miRNA transcriptome is represented as movement in the potential. In normal control mice in the absence of an oncogenic event, there exists a large energy barrier between a state of normal hematopoiesis and AML, corresponding to a low probability of state-transition (left). An oncogenic event, such as *Cbfb-MYH11* (*CM*) fusion resulting from inv(16), alters the quasi-potential, reducing the energy barrier and increasing the probability of state-transition (right). The states are characterized by local maxima and minima, labeled *c*_1_, *c*_2_, and *c*_3_, corresponding to perturbed hematopoiesis, an unstable transition, and AML states, respectively. (**B**) The first two PCs (PC1 and PC2) of a time-series study of miRNA expression in a murine model of *CM*-driven AML reveals state-transition trajectories that are mapped to critical points in the model.

We performed a time-series miRNA sequencing study using a conditional *Cbfb-MYH11* (*CM*) knock-in mouse model (*Cbfb^+/56M^/Mx1-Cre;* C57BL/6), which recapitulates human inv(16) AML (*4*–*7*). Using a state-transition model, we represented the miRNA transcriptome of each mouse as a particle undergoing Brownian motion in a quasi-potential with two stable steady states: perturbed hematopoiesis (*c*_1_) and AML (*c*_3_) separated by an unstable transition state (*c*_2_) (Fig. 1A; see Materials and Methods for mathematical details). We hypothesized that induction of the leukemogenic *CM* gene would perturb the quasi-potential such that a transition to AML would become more likely. To test this hypothesis, we performed principal components analysis (PCA) on the time-series miRNA transcriptome data with the singular-value decomposition (SVD) to construct an AML state-space, where critical points of the leukemogenic potential could be mapped and where state-transition trajectories could be visualized. Each PC produced by the SVD was correlated with *Kit* mRNA expression and the percent (%) of cKit^+^ cells detected by flow cytometry, as cKit^+^ cells represent AML blasts that progressively increase during leukemogenesis in this model (figs. S1 and S2). We identified PC1 as the PC that most strongly correlated with *Kit* expression [coefficient of determination (*R*^2^) = 0.68; *P* < 0.001; fig. S2 and table S1] and resulted in the largest separation between *CM* and control samples. We therefore used PC1 to define the miRNA AML state-space.

**Fig. 2.**
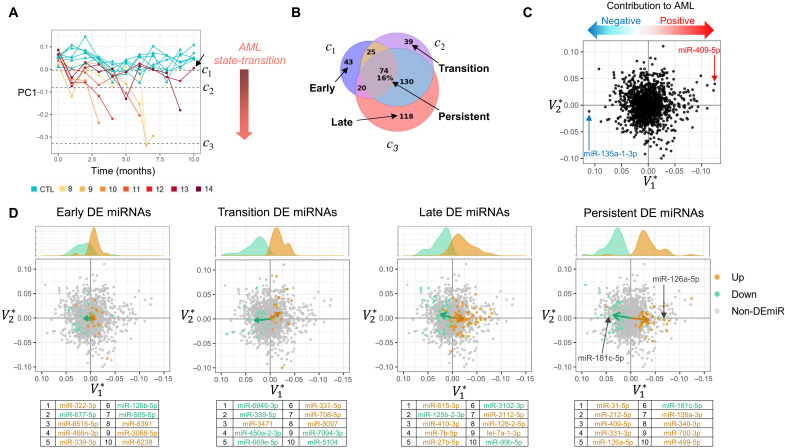
Critical point–based differential expression analysis. (**A**) The miRNA transcriptome state-transition and critical points are visualized by plotting the first PC (PC1) over time. Trajectories of *CM* and control mice diverge over time as the value of PC1 becomes more negative as *CM* mice manifest AML and move toward the AML state (*c*_3_). *CM* mouse 13 did not develop AML and did not cross the transition critical point *c*_2_ (black arrow). (**B**) Critical points in the state-transition model are used to guide differential expression analysis and to define early, transition, late, and persistent differential expression events as compared to control samples. Numbers indicate the number of miRNAs differentially expressed. (**C**) PC loadings ($V_{1}^{*}$, $V_{2}^{*}$) provide a geometric interpretation of miRNA expression so that miRNA may be identified as having a positive or a negative contribution to the construction of the AML state-space. (**D**) Early, transition, late, and persistent DE miRNAs shown in the state-space and are colored for up- or down-regulation. The sum total contribution of DE miRNA in each event is visualized as a vector calculated by the mean of all DE miRNAs. The magnitude of the $V_{1}^{*}$ component of the vector indicates how strongly the DE miRNA contributes to AML state-transition (i.e., sample movement toward AML in the state-space). Early events have smaller contribution to AML, revealed by small $V_{1}^{*}$ component, followed by increasing contributions to AML in transition events, with larger contributions in late and persistent events. Kernel density plots show the distribution of $V_{1}^{*}$ values for up- and down-regulated DE miRNAs. Tables indicate the 10 most significantly DE miRNA in each comparison.

Notably, as early as 1 month after induction, and at the two subsequent time points, differences in PC1 between *CM* and control samples could be detected, before any evidence of change in the percentage of circulating cKit^+^ blasts as detected by flow cytometry (see fig. S3 and table S2). To map state-transition trajectories and critical points for each mouse, we plotted time-sequential samples of *CM* and control mice and observed that as the *CM* mice developed AML, their PC1 value decreased in a manner consistent with the state-transition model (Fig. 1B, figs. S4 and S5, and tables S3 and S4). In particular, six of seven *CM*-induced mice developed AML during the 10-month duration of the study, and all six *CM* mice that developed AML died after crossing the critical point *c*_2_. At the end of the experiment, the only one CM mouse that did not develop AML had not crossed the critical point *c*_2_ and had no detectable circulating cKit^+^ blasts in the peripheral blood (Fig. 2A and figs. S1B and S6; mouse 13).

### State-transition critical points enable investigation of differentially expressed miRNA

Although the time-series sampling allowed us to observe changes in miRNA expression during leukemia initiation and progression, the *CM* mice did not develop leukemia at the same time (Fig. 2A). Consequently, *CM* mice were at different states of AML development at any given fixed time point. Therefore, to identify differentially expressed miRNAs (DE miRNAs), we used the state-space and state-transition critical points as pseudo-time points to align phenotypic states of disease among individual mice. Pairwise comparisons of miRNA expression at each critical point between the *CM* and control mice allowed us to identify early, transition, late, and persistent DE miRNAs occurring during AML progression (*8*). Early events were defined as the unique DE miRNAs that occurred at *c*_1_, transition events were those that occurred at *c*_2_, and late events were those that occurred at *c*_3_, while persistent events were DE miRNAs at all three critical points (*c*_1_, *c*_2_,and *c*_3_; Fig. 2B and table S5).

To connect DE miRNAs to the AML state-space, we compared the PCA loadings (i.e., V^*^ the right singular vectors of the SVD) to the PC values (Fig. 2C). The contribution of each DE miRNA to the AML state-space was determined by its loading value associated with the state-space component PC1 ($V_{1}^{*}$). Thus, each miRNA had either a positive or negative contribution to the AML state-space construction determined by its $V_{1}^{*}$ loading value. The contribution of each miRNA to AML state-transition depended on both its $V_{1}^{*}$ loading value and whether it was up- or down-regulated. In particular, an miRNA could contribute to movement in the state-space and therefore to AML state-transition with either (i) a negative $V_{1}^{*}$ loading value and increased expression or (ii) a positive $V_{1}^{*}$ loading value and a decrease in expression. For example, miR-409-5p had the largest negative loading value and was up-regulated and, therefore, had the largest contribution to AML in the state-space because progression to AML was associated with negative PC1 values. In contrast, miR-135a-1-3p had the largest positive loading value, but it decreased in expression; therefore, it also had a positive contribution to AML in the state-space.

To summarize the net contribution of DE miRNA in early, transition, late, and persistent events, we plotted vectors representing the mean of the miRNA loadings of up- and down-regulated DE miRNA [$\vec{v}=(V_{1}^{*},V_{2}^{*})$; Fig. 2D]. The magnitude of the $V_{1}^{*}$component of the vector was used to quantify the contribution to AML state transition. In particular, two persistent DE miRNAs (i.e., their expression change was statistically significant compared to controls at all time points) illustrate strong contributions to AML: The expression of miR-126a, previously shown to support inv(16) AML growth, progressively increased (*9*); and the expression of miR-181c, which was shown to be involved in hematopoietic differentiation (*10*, *11*), progressively decreased. When we applied this interpretation to the early, transition, late, and persistent DE miRNAs, we observed that early events had a smaller net contribution to AML as compared to the transition or late events. To interrogate the biological role of the DE miRNAs constituting these events, we identified pathways implicated by early, transition, late, and persistent DE miRNAs (*12*). Early events involved cytokine signaling and inflammatory pathways; transition events involved Wnt signaling and metabolic pathways; and late events involved Kit, mitogen-activated protein kinase (Mapk), p53 signaling pathways, and persistent events involved apoptosis, adhesion, and phosphatidylinositol 3-kinase (PI3K)–Akt signaling pathways (fig. S7).

### Dynamics of miRNA expression using AML state-space critical points

**Fig. 3.**
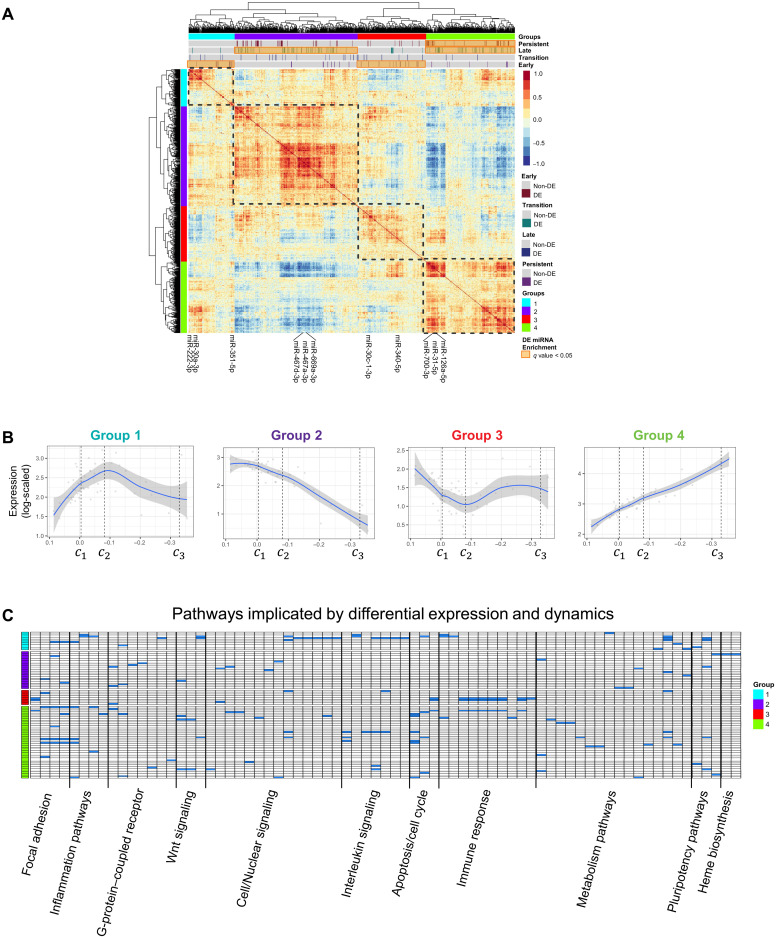
Expression dynamics of miRNA in AML state-space. (**A**) Hierarchical clustering of correlated miRNA expression annotated with state-transition critical point–based DE miRNAs. Four distinct patterns of miRNA expression are identified. (**B**) Four patterns, or groups of miRNA expression dynamics include monotonic (groups 2 and 4) and nonmonotonic (groups 1 and 3) patterns when plotted in the state-space (PC1). These patterns were discovered by fitting the average log-scaled expression for each sample in each expression group when plotted as a function of the PC1 coordinate of each sample. A local maximum (group 1) and minimum (group 3) are identified near the unstable transition critical point *c*_2_. (**C**) Pathways implicated by the nonmonotonic expression pattern in group 1 include cell/nuclear signaling pathways including IL-6, TNFα-NFκB, cytokine inflammation response, and Wnt signaling. The opposite expression pattern (group 3) is enriched for immune response pathways, including antigen processing and TLR signaling (complete list provided in table S7.)

In addition to differential expression and pathway analysis, we investigated miRNA expression dynamics with correlation analysis and the state-transition critical points. Hierarchical clustering of the correlation coefficients between all pairs of miRNA revealed four distinct groups of expression dynamics (Fig. 3A and table S6). When plotted in the state-space (PC1), the four groups corresponded to monotonic and nonmonotonic expression patterns (Fig. 3B). The miRNA with nonmonotonic expression dynamics revealed a local maximum (group 1) or local minimum (group 3) around the critical point *c*_2_. As *c*_2_ is the critical point in the state-transition model that separates perturbed hematopoiesis and overt AML, changes in miRNA expression at or near *c*_2_ suggested that these miRNAs may play a role in facilitating an irreversible transition to AML.

Notably, when we tested each dynamic expression group for overrepresentation of early, transition, late, or persistent DE miRNAs, we observed that the monotonic expression groups showed overrepresentation of late and persistent events, whereas the nonmonotonic expression groups showed overrepresentation of early events. Pathway analysis revealed that the nonmonotonic miRNA expression dynamic pattern in group 1 was associated with cytokines and inflammatory response [e.g., Wnt, interleukin-6 (IL-6), tumor necrosis factor–α (TNFα)–nuclear factor κB (NFκB), and transforming growth factor–β signaling], while group 3 was associated with immune response pathways including antigen processing and Toll-like receptor (TLR) signaling (Fig. 3C; pathway summary in table S7). Similar to group 3, group 4 was also associated with immunogenic and TLR signaling but differed from group 3 in its association with Mapk and PI3K-Akt signaling. Group 2 involved more heterogeneous pathways, which included metabolism, p53 signaling, and adhesion pathways.

### Validation of miRNA state-space in an independent cohort

**Fig. 4.**
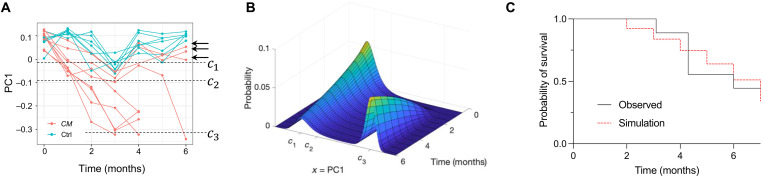
Validation of the AML state-space and state-transition model. (**A**) Using only the state-space PC loadings $V_{1}^{*}$, samples from an independent cohort of *CM* and control mice were projected into the state-space (*CM*, *n* = 9; control, *n* = 7). Control and *CM* state-transition trajectories were accurately identified, including three *CM* mice that did not develop leukemia at the experiment end point (black arrows). (**B**) The solution of the FP model of state-transition gives the evolution of the probability of the miRNA transcriptome particle being at a point in the state-space at any given time. (**C**) The solution of the FP equation with parameters derived from the training cohort is integrated over the state-space past the transition point *c*_2_ to calculate the probability to develop AML over time. Using a Gaussian kernel with the initial state of the miRNA transcriptome following *CM* induction [*t* = 1 shown in (A)], the FP equation accurately predicts AML in the validation cohort of *CM* mice, including the prediction that not all mice would manifest AML in the 6-month duration of the experiment. The log-rank test was unable to reject the null hypothesis that there is no difference between the observed and simulated curves [*P* = 0.79, HR = 0.86 (0.26 to 2.8)].

As a validation of the miRNA-based AML state-space, the loading values ($V_{1}^{*}$) were used to project data from an independent experiment of *CM* and control samples (validation cohort) into the state space (see Supplementary Text). Without any prior knowledge of the genotype or time point of the samples, the PC1 loadings ($V_{1}^{*}$) predicted the disease status and trajectories of the new samples (Fig. 4A). Notably, the state-space trajectories of three *CM* mice in the validation cohort that were induced but did not develop AML during the observation period (6 months) were correctly projected to be with the control samples and did not cross the transition critical point *c*_2_ (Fig. 4A; black arrows).

### The state-transition model correctly predicts time to AML development

Thus far, we have illustrated the use of critical points as pseudo-time points to phenotypically synchronize the transition of each mouse from perturbed hematopoiesis to AML. However, the state-transition model can also be used to predict AML development in each mouse in chronological time. To predict the time to develop AML, we used a Fokker-Planck (FP) probability density equation related to the stochastic equation of motion in the quasi-potential (Eq. 1). We solved the FP equation forward in time with parameters estimated from the training cohort of *CM* mice with a Gaussian initial condition based on the first time point after induction of *CM* samples in the state-space from the validation cohort (Fig. 4B and fig. S8). By integrating the solution of the FP from *c*_2_ to *c*_3_, we calculated the probability of state-transition over time. We then compared the predicted time to develop AML with that observed in validation cohort of *CM* mice (*n* = 9) using a survival analysis. Our model accurately predicted the time to develop AML, as the predicted and observed survival curves were not statistically different [log-rank, *P* = 0.79; hazard ratio (HR) = 0.86 (0.26 to 2.8); Fig. 4C]. Notably, the model correctly predicted that not all mice would manifest AML during the 6-month duration of the experiment.

### miRNA and mRNA transcriptomes both encode AML state-transition

**Fig. 5.**
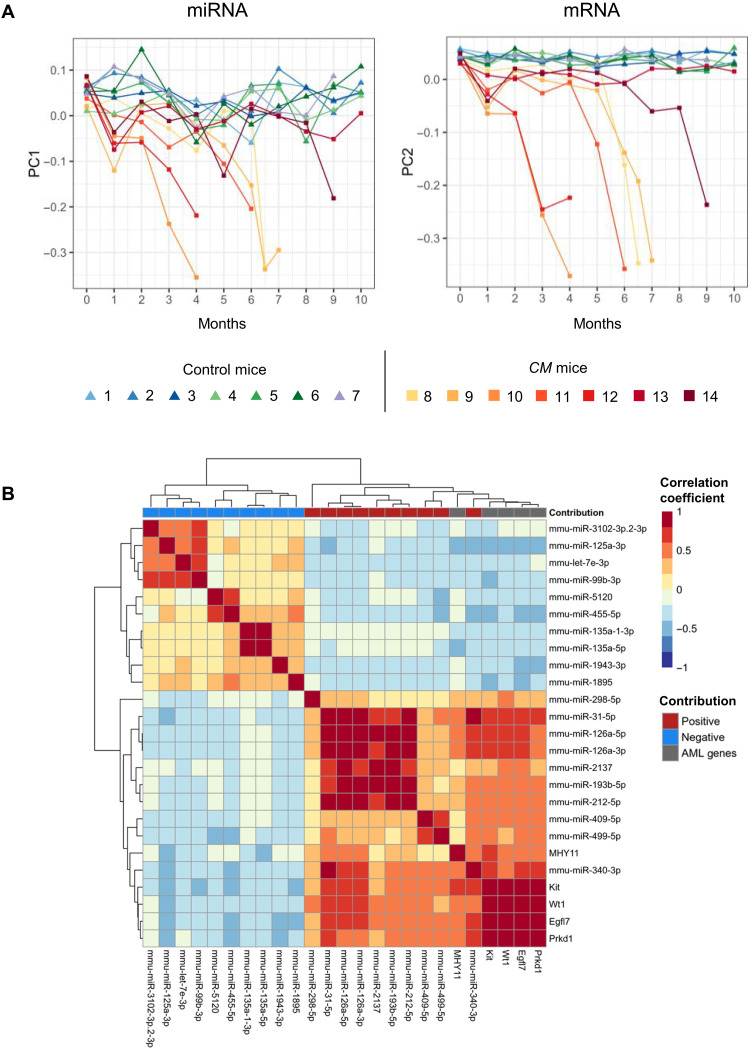
Comparison of miRNA and mRNA-derived AML state-spaces. (**A**) AML state-transition trajectories from miRNA and mRNA state-spaces over time reveal similarities in dynamics for individual *CM* mice. (**B**) Hierarchical clustering of a correlation matrix constructed with time-series expression of five AML-promoting genes identified in our previous work (*Prkd1*, *Egfl7*, *Wt1*, *Kit*, and *Cbfb-MYH11*) and persistent DE miRNAs that had the largest positive and negative contribution to AML. Positive and negative correlations between miRNAs and mRNA genes involved in AML state-transition are consistent with the state-space and mathematical model predictions. The largest positive correlation between the miRNAs and the five mRNA genes was between the miRNA that had a positive contribution to AML and the AML-promoting genes.

Because we have previously shown that the mRNA transcriptome (RNA sequencing) undergoes state-ransition during leukemogenesis using data from the same mouse model (*13*), we compared the mRNA and miRNA-based state-spaces and trajectories to each other. To quantitatively assess the similarity of mRNA- and miRNA-defined state-spaces, we computed the angle between all the miRNA and mRNA PC pairs using the vector dot product (see Supplementary Text). We interpreted the angle between the vectors as follows: miRNA and mRNA PCs that had an angle close to zero encoded similar sources of variance. Notably, the two PCs that were most similar were those that defined the AML state-space (i.e., PC1 for miRNA and PC2 for mRNA; fig. S9A). Notably, not only were no other PC pairs more aligned than miRNA PC1 and mRNA PC2, but almost all other PCs were nearly orthogonal to each other. By plotting the miRNA and mRNA state-spaces against each other and annotating the critical points, the overall state-transition trajectories had a notable similarity (Fig. 5A and fig. S9B). Only 5 of 129 total samples were classified differently as being in *c*_1_, *c*_2_, or *c*_3_ states based on which critical points—mRNA or miRNA—were used. This suggests that the time-series expression dynamics of miRNA and mRNA transcriptomes can be used to predict AML development because they encode similar, but not identical, information of AML state-transition.

To examine the similarity in expression dynamics between miRNA and mRNA further, we calculated correlation coefficients between miRNAs and five mRNAs identified to have a large contribution to AML in our previous work (*Prkd1*, *Egfl7*, *Wt1*, *Kit*, and *Cbfb-MYH1l*) (*13*) and performed hierarchical clustering on the correlation matrix. To focus our analysis, we selected the persistent DE miRNAs that had the largest absolute PC1 loading value ($V_{1}^{*}$) for comparison to the five AML-promoting genes (Fig. 5B). The miRNAs with positive contribution to AML were most correlated with the AML-promoting genes; conversely, the miRNAs that have a negative contribution to AML were negatively correlated. Among the miRNAs that had strong positive correlation, miR-126a was highly correlated with all five AML-promoting genes including its host gene *Egfl7*, consistent with its functional contribution to inv(16) AML development (*9*). Our results predict that the other highly positively correlated miRNAs including miR-31 and miR-340, which either have not been previously reported or have complex and sometimes conflicting roles in cancer development (*14*–*17*), also have a strong contribution to inv(16) AML. Together, the similarity of the state-spaces, critical point locations, and expression dynamics between the miRNA and mRNA demonstrated that both miRNA and mRNA expression undergo a system-wide state-transition during AML development and they each encode information of disease progression that is similar, but not identical for leukemia state-ransition.

## DISCUSSION

Here, we report the application of a state-transition model and theory to the interpretation of how temporal changes in bulk miRNA expression in the peripheral blood informs AML initiation and progression. The state-transition model is a system-wide holistic approach to biology where the transformation from one state to another is viewed as a change in state of the whole system as opposed to a change resulting from one (or a small collection) of molecules (*18*–*20*). Using miRNA expression as an example of this perspective, a state-transition occurs because the entire miRNA transcriptome transitions to a new steady state: The expression of all miRNAs contribute to the transition, not the change in expression of a single miRNA. To be clear, as was the case in our *CM* AML mouse model, a single mutation or molecule may be sufficient to cause a perturbation that induces a state-transition; however, in the system-wide holistic view, the perturbation causes an alteration to the underlying regulatory network that results in a state-transition to the entire transcriptional state.

Dimensionality reduction techniques, such as the SVD used here and the generalized SVD, have proven useful for multiomic and pan-cancer studies because they decompose the information in the data into linear combinations of orthogonal basis vectors (*13*, *21*–*24*). Similar mathematical approaches, including endogenous network theory, have been used to identify steady states, or attractors, using configurations of gene regulatory networks (*2*, *19*, *25*–*30*). We compared state-spaces constructed with other methods including diffusion map, t-distributed stochastic neighbor embedding (t-SNE), and uniform manifold approximation and projection (UMAP) with that constructed with the SVD. The SVD was used in the final analysis because it produced the largest separation between *CM* and control samples and because it is a linear method, with no adjustable hyperparameters (fig. S10). Our finding that the miRNA transcriptome can be used to construct an AML state-space and corresponding predictive state-transition model is, to the best of our knowledge, the first report of miRNA transcriptome encoding system-wide dynamics during AML pathogenesis and progression. Thus, this work reveals that a more system-wide holistic view of the miRNA transcriptome is warranted.

The state-space and state-transition model provided a theory-guided approach to the analysis of differential expression, with early, transition, late, and persistent events defined relative to the state-transition critical points *c*_1_, *c*_2_, and *c*_3_. This, in turn, allowed for novel quantifications of miRNA contributions to AML pathogenesis through identification of distinct dynamics of miRNA expression, including monotonic and nonmonotonic patterns. The miRNAs with monotonic patterns of expression, i.e., continuously decreased or continuously increased expression, were enriched with miRNAs that were found to be persistent DE miRNAs throughout AML progression at all three critical points. These groups included miRNAs that regulate genes involved in the “inflammasome” (i.e., miR-467), cell differentiation (i.e., miR-669 and miR-31), and leukemia stem cell function (i.e., miR-126). Consistent with our finding that miR-126 is a top persistent DE miRNA with continuously increased expression, miR-126 has been reported as a hallmark of inv(16) AML (*31*). To this end, we have recently demonstrated that aberrant miR-126 overexpression is crucial for *CM*-induced AML development (*9*). In addition, several miRNAs (i.e., miR-100-5p, miR-142-3p, and miR-99-3p) previously reported in association with inv(16) AML are consistent with our predicted contribution to AML and show monotonic patterns of expression (table S8) (*9*, *31*–*33*). These findings highlight the potential biological relevance of our results and support our hypothesis that state-transition–guided analysis of differential miRNA expression may provide information that could otherwise be missed.

We interpreted monotonic miRNA expression in the context of our state-transition model as a representation of a “leukemogenic force” given by the continuous increase—or decrease—of onco- and tumor-suppressor miRNAs during AML state-transition. In contrast, we interpreted the nonmonotonic miRNA expression dynamics as a representation of a “restoring force” that attempts to return the system, represented by miRNA transcriptome, to an equilibrium state (i.e., perturbed hematopoiesis, or *c*_1_) after *CM* induction but that inevitably breaks down at the “point of no return” in AML state-transition (i.e., the transition critical point *c*_2_), at which point it is overcome by the leukemogenic force. The miRNA groups with nonmonotonic expression patterns included several miRNAs that regulate glucose and lipid metabolisms (i.e., miR-320 and miR-142) or directly target *Kit* and *Pten* (i.e., miR-222) or ubiquitination (i.e., miR-378 and miR-30c). This interpretation of miRNA expression dynamics suggests that the miRNA transcriptome may encode critical information about the state of the system as a whole and may serve as a novel blood-based biomarker of state-transition to AML or as an early indicator of response to a therapeutic intervention. Without the AML state-space and state-transition critical points, the nonmonotonic expression patterns of groups 1 and 3 would not be interpreted in this way.

To confirm the reproducibility and predictive value of the miRNA-derived state-space, we applied the model to similar time-series miRNA sequencing data from an independent cohort of mice. The state-space correctly differentiated *CM* and control samples and state-transition trajectories. Once the validation cohort was projected into the state-space, we used the state-transition mathematical model to predict the time to develop AML and found no statistically significant difference between the predicted and actual survival curves for the validation cohort of mice, supporting the accuracy of our state-transition model prediction.

Although we investigated and reported pathways implicated by miRNA expression patterns and by DE miRNAs, detailed analyses were limited because of the heterogeneous composition of cells in the bulk expression data taken from peripheral blood, combined with limited miRNA-gene target regulatory interactions reported in mice. While the biological meaning of miRNA expression dynamics warrants further investigation both in silico and at the bench, we believe that these patterns represent important features of leukemogenesis and progression. Regardless, we provide here the proof of concept that state-transition theory can describe and predict AML evolution in a mouse model. Whether the inv(16) AML state-space is specific for this cytogenetic subtype or is also applicable to other AML subtypes remains to be determined. It would be quite unlikely that inv(16) is so unique as to the state-transition model not being generalizable to other subtypes of AML. To further demonstrate the translational applicability, we are currently working to apply our approach to analyze human data.

Notably, we recently showed that state-transition model and theory can also predict AML initiation and development of the mRNA transcriptome (*13*). When we analyzed mRNA- and miRNA-derived state-spaces, state-transition trajectories, the angle between the leukemia-associated PCs, and the locations of the critical points, we observed that both miRNA and mRNA expression undergo a system-wide state-transition during AML development and that they encode information of disease progression that is similar, but not identical. Thus, while miRNAs and mRNA are mostly functionally associated, it is possible that certain steps of leukemogenesis and progression are likely uniquely dependent on either miRNA or mRNA expression. We expect that simultaneous state-transition modeling of mRNA and miRNA expression dynamics will provide a unique perspective to map the interrelationships and information content that, applied collectively, will be instrumental to detect early indications and monitor treatment response or predict relapse in individual patients with AML.

## MATERIALS AND METHODS

### Experimental design

Our study was designed to construct a state-transition model on AML development using a well-established mouse model of AML. We used two independent cohorts of mice as training and validation cohorts. For the training cohort, we collected PBMC from the *CM*-induced mice (*n* = 7) and the similarly treated littermate controls (*n* = 7) before induction (*t* = 0) and monthly after induction up to 10 months (*t* = 1 to 10) or when the mouse developed leukemia and became moribund, whichever event occurred first. Similarly, for the validation cohort, we collected PBMC from *CM* mice (*n* = 9) or control (*n* = 7) before induction and monthly thereafter up to 6 months. Total RNA was isolated from PBMC using the AllPrep DNA/RNA Kit (Qiagen). Sequencing details including library preparation and differential expression analysis can be found in the Supplementary Text.

### Mouse model

The expression of the leukemogenic fusion gene *Cbfb-MYH11* (*CM*) in conditional *CM* knock-in mice (*Cbfb^56M/+^/Mx1-Cre*) leads to development of AML with a median survival of approximately 4 months after induction of *CM*. This model recapitulates human inv(16) AML, one of the common subsets of AML characterized by the rearrangement of chromosome 16 at bands p13 and q22, which, at the molecular level, creates the chimeric fusion gene *CBFB-MYH11*. To induce *CM* expression, 6- to 8-week-old *CM* knock-in mice were intraperitoneally injected with polyinosinic–polycytidylic acid [poly (I:C)] (InvivoGen, tlrl-picw-250) at 14 mg/kg per dose every other day for a total of seven doses. Age-matched littermates lacking the transgene were similarly treated and used as control. All mice were maintained in an Association for Assessment and Accreditation of Laboratory Animal Care–accredited animal facility, and all experimental procedures were performed in accordance with federal and state government guidelines and established institutional guidelines and protocols approved by the Institutional Animal Care and Use Committee at City of Hope.

### Mathematical model of state-transition

Using a state-transition model, we represented the miRNA transcriptome of each mouse as a particle in a double-well quasi-potential with two stable steady states: perturbed hematopoiesis (*c*_1_) and AML (*c*_3_) separated by an unstable transition state (*c*_2_). After induction of the leukemogenic *CM* gene, we postulated that the potential energy landscape was perturbed such that a transition from a state of perturbed hematopoiesis to leukemia became more likely.

We modeled the miRNA state-transition trajectories for individual mice as a particle undergoing Brownian motion in the double-well quasi-potential energy with a Langevin equation of the form $dX_{t}=-\nabla U_{p}(X_{t})\mathrm{dt}+\sqrt{2\beta^{-1}}dB_{t}$. The position of the particle in the quasi-potential is denoted *X_t_*, *B_t_* is a Brownian stochastic process that is uncorrelated in time 〈*B_i_*, *B_j_*〉 = δ_*i*, *j*_, and the double-well quasi-potential *U_p_* is given by a polynomial specified by critical points (*c*_1_, *c*_2_, *c*_3_) that correspond to the local minima and maxima of the quasi-potential, so that *U_p_*(*x*) = α ∫ (*x* − *c*_1_)(*x* − *c*_2_)(*x* − *c*_3_)*dx*, where α is a scaling parameter.

To be precise, we use the term quasi-potential to specify that this is a model and not a physical energy potential. We defined the double-well quasi-potential with a polynomial because this is a simple and parsimonious mathematical interpretation of our model, assuming perturbed hematopoiesis and AML to be stable stationary states. This assumption relies on the fact that in absence of an oncogenic event, the probability of spontaneous transition to AML is low, and conversely, in a state of AML, in absence of treatment, the probability of transitioning back from AML to normal or perturbed hematopoiesis is also low. Although many states of the system may exist, the aim of our model was to capture state-transition dynamics between two clearly defined phenotypic states observable in our mouse model: perturbed hematopoiesis and AML.

Because the Langevin equation of motion is a Brownian stochastic process, the probability distribution for a miRNA transcriptome particle to be at a certain position at a given time is given by an FP equation (fig. S8). Thus, to compute the probability of state-transition at a point in state-space and time *P*(*x*, *t*), we solved the FP equation related to the stochastic equation of motion, given by

$$\frac{\partial }{\partial t}P(x,t)=-\frac{\partial }{\partial x}(\nabla U_{p}(x)P(x,t))+\frac{\partial^{2}}{\partial x^{2}}\beta P(x,t)$$ (1)

where β is the diffusion coefficient (and is the same parameter found in the Langevin equation) and *U_p_*(*x*) is the quasi-potential. The diffusion coefficient was estimated for the *CM* and control mice as the average slope of the mean-squared displacement in the state-space over time (fig. S11).

### Constructing the AML state-space

To construct a miRNA-based state-space required to describe AML state-transition, we used the SVD to perform PCA on the data matrix (X). The data matrix was composed of all time-sequential samples from *CM* and control mice as rows and miRNAs as columns, so that *X* = *U*ΣV^*^ where the columns of *X* were mean-centered, log-normalized counts and * indicates the conjugate transpose. The SVD therefore decomposed the log-transformed normalized miRNA count matrix into singular values and left- and right-singular vectors (*U* and V^*^, respectively). PC1was identified as the component that was most strongly correlated with expression of the immunophenotypic marker gene Kit and resulted in the greatest separation between *CM* and control samples and therefore was used to define the state-space. PC2 was chosen as a second component to visualize a two-dimensional state-space for simplicity and convenience; because each PC is orthogonal to each other by construction, any other PC would create an orthogonal two-dimensional space.

The singular vectors are basis vectors of the miRNA transcriptome and span either the state-space (*U*; composed of samples) or the feature space (V^*^; in this context, the feature space contains the miRNA loadings). Therefore, singular vectors or linear combinations of singular vectors represent lower dimensional representations of the miRNA transcriptome. In addition, each singular vector has an associated singular value that indicates what fraction of the total variance is explained by the associated singular vector. The singular values were ordered from largest to smallest. The associated left-singular vectors (columns of U) corresponded to the state-space, and the PCs are given as PC = *U*Σ. The right singular vectors (V^*^) are the PC loadings (i.e., the coefficient weights of miRNA contributions to the PCs; table S9).

### Mapping the critical points of the AML double-well quasi-potential

Critical points and AML state-transition dynamics in the state-space were identified with PC1 and used to define states of perturbed hematopoiesis, transition, and overt AML (Fig. 1B). Following our previous work, *K*-means clustering with *K* = 3 was performed on the PC1 coordinates of the *CM* samples to identify the three critical points (*c*_1_, *c*_2_, and *c*_3_) that define the double-well quasi-potential (*U_p_*) (*13*). The critical points *c*_1_ and *c*_3_ were taken as the means of the clusters including the perturbed early time points and AML samples, denoted K1 and K3, respectively. The transition critical point *c*_2_ was estimated by maximizing the Boltzmann ratio between the *c*_1_ and *c*_3_ states as $c_{2}=\text{arg}\;{\text{max}}_{x\in K2}e^{-(U_{p}(x,c_{3})-U_{p}(x,c_{1}))k_{B}}$, where K2 is the cluster between K1 and K3 and *k*_B_ is the Boltzmann constant (fig. S4 and table S4). Simulation studies confirmed that the cluster means of K1 and K3 were the best estimators of *c*_1_ and *c*_3_, respectively, and *c*_2_ was best estimated to be near the boundary of clusters K1 and K2 (fig. S5).

### Validation analysis

The conjugate transpose of the right singular vectors of the SVD (*V*) were used to project data from the validation cohort matrix (*Y*) into the state-space constructed with the training cohort as *YV* = *U_Y_*Σ*_Y_* with the intersection of miRNA from the training and validation data (Fig. 4A). The validation state-space was taken as the first component of this projection, consistent with the training data. Critical points were the same for validation as training.

To predict the time to develop AML, parameters derived from the training data were used to solve the FP equation using a Gaussian kernel initial condition derived from the validation cohort data (fig. S11 and table S4). The predicted time to AML was calculated by integrating the probability density over time from the critical point *c*_2_ to negative infinity and was evaluated at the experimental observation points to create a survival curve, which was compared to the observed survival of the mice in the validation cohort. The log-rank test was used to test the null hypothesis that there was no difference between the observed and simulated survival curves at any time point. The log-rank test was performed in Prism Graphpad 9.2.0 and was unable to reject the null hypothesis with *P* = 0.79, and HR = 0.86 (0.26 to 2.8).

## Supplementary Materials

### This PDF file includes:

Supplementary Text Figs. S1 to S11 Tables S2, S4, and S8 References

## Notes

View/request a protocol for this paper from *Bio-protocol*.
