# Supplementary material for: Dynamic patterns of microRNA expression during acute myeloid leukemia state-transition
Source: Sci Adv. 2022 Apr 22;8(16):eabj1664. doi: 10.1126/sciadv.abj1664 (PMC9032952; doi:10.1126/sciadv.abj1664)
Supplement: Supplementary file 1 — Supplementary Text Figs. S1 to S11 Tables S2, S4, and S8 References [file sciadv.abj1664_sm.pdf]

Science Advances
AAAS

Supplementary Materials for

Dynamic patterns of microRNA expression during acute myeloid leukemia state-transition

David E. Frankhouser, Denis O'Meally, Sergio Branciamore, Lisa Uechi, Lianjun Zhang, Ying-Chieh Chen, Man Li, Hanjun Qin, Xiwei Wu, Nadia Carlesso, Guido Marcucci*, Russell C. Rockne*, Ya-Huei Kuo*

*Corresponding author. Email: ykuo@coh.org (Y.-H.K.); rrockne@coh.org (R.C.R.); gmarcucci@coh.org (G.M.)

Published 22 April 2022, *Sci. Adv.* **8**, eabj1664 (2022)
DOI: 10.1126/sciadv.abj1664

The PDF file includes:

Supplementary Text
Figs. S1 to S11
Tables S2, S4, and S8
Legends for tables S1, S3, S5, S6 and S7, S9 to S12
References

Other Supplementary Material for this manuscript includes the following:

Tables S1, S3, S5, S6 and S7, S9 to S12

Supplementary Text

miRNA-seq library preparation and sequencing

Testing cohort samples were allocated to randomized batches for library preparation, such that samples from each timepoint were distributed evenly over all sequencing runs. Validation cohort samples were processed in batches as collected. All libraries were prepared using the Illumina TruSeq Small RNA protocol with minor modification following the manufacturer's instructions. Briefly, for each sample, 280 ng of total RNA was ligated to the sRNA 3′ adaptor (5'-TCTGGAATTCTCGGGTGCCAAGGAACTCC-3') with T4 RNA Ligase 2, truncated (New England BioLabs) for 1 h at 22°C, and subsequently ligated to a 5′ adaptor: 5'-GUUCAGAGUUCUACAGUCCGACGAUCNNN-3') with T4 RNA ligase 1 (New England BioLabs) for 1 h at 20°C. The constructed small RNA library was first reverse-transcribed using GX1 (5′- GGAGTTCCTTGGCACCCGAGA) as the RT primer then subjected to PCR amplification for 13 cycles, using the primers GX1 (5′-CAAGCAGAAGACGGCATACGAGAT[NNNNNN]GTGACTGGAGTTCCTTGGCACCCGAGAATTCCA-3') and GX2 (5′-AATGATACGGCGACCACCGAGATCTACAC[NNNNNNNN]CGACAGGTTCAGAGTTCTACAGTCCGA-3'), followed by 6% TBE PAGE gel purification with size selection (for targeted small RNAs of 17–35 nt). Individual libraries were prepared using a unique index primer (NNNNNNNN in the GX1 and GX2 primer) in order to allow for pooling of multiple samples prior to sequencing. The purified libraries were quantified using qPCR. Sequencing of paired- (testing cohort) or single-end (validation) 50 cycles was performed on a HiSeq 2500 (Illumina Inc., San Diego, CA), and image processing and base calling were conducted using Illumina's RTA pipeline.

Raw sequencing reads were processed with the nf-core smRNASeq pipeline version 1.0(*34*) using the GRCm38 genome reference (with the parameter --genome GRCm38) and adapters for the Illumina small RNA protocol (by setting --protocol illumina). Briefly, trimmed reads were mapped using bowtie(*35*) to miRBase(*36*) mature miRNAs (using the parameters -k 50 --best --strata), and the number of reads mapping to each was counted using samtools stats*(37*). Each library was also subjected to extensive quality control, including estimation of library complexity, contamination, sequence quality, read length and depth, among other metrics detailed in the pipeline repository. Mapped reads were merged into a matrix of counts per gene for each sample at each timepoint and normalized to counts per million (CPM) reads mapped, as implemented in edgeR(*38*). Surrogate variable analysis was used to check for confounding experimental effects(*39*). None was apparent in the testing set. The miRNA dataset is submitted to GEO under accession number GSE173785.

miRNA analysis

Log normalized miRNA were generated by taking the log (base 2) of CPMs [i.e., log2(CPM+0.01)] and used for singular value decomposition (SVD). We treated each mouse as a replicate to investigate how expression changed as the mice moved through the leukemic state-space (PC1). Differentially expressed (DE) miRNA were determined by comparing control samples to the samples classified according to critical points ($c_1, c_2,$ and c_3) using miRTOP generated miRNA counts and default settings of DEseq2(*40*, *41*). For the validation cohort, data were processed by removing adapters using cutadapt v1.9.1, and trimmed sequences were aligned to mm9 genome using Bowtie v0.12.7 with "--best" option(*42*, *43*). Mature miRNAs

counts were determined using R scripts and miRbase v21(*36*). Log normalized counts were again generated from CPM [i.e., log2(CPM+0.01)]; one sample (out of 99 total samples) was removed as an outlier based on poor library quality and abnormal expression patterns.

Identification of the miRNA state-space and correlation with Kit expression
In order to identify which principal component was most associated with AML state-transition, we examined all principal components of the data matrix composed of all time-series miRNA expression data from CM and control samples and correlated them with expression of *Kit* gene, which is an immunophenotypic marker of AML. We observed that PC1 had both the highest R^2 correlation with Kit expression as well as the lowest p-value of all PCs (**Figure S3, Table S1**). PC1 and PC2 accounted for 5 and 4%, respectively, of the total variance present in the data. Kit expression was determined using the matched mRNA sequencing (RNA-seq) for each sample as previously described(*13*). The percentage of cells that expressed the cKit protein (% cKit+) was determined in each sample using flow cytometry (**Table S10**). cKit+ was compared to PC1 and time (**Figure S1A-B**) and was shown to be similar to Kit mRNA expression (**Figure S1C**). To confirm that the miRNA state-space loadings (V_1^*) were associated with AML, we calculated the correlation between each miRNA expression and % cKit+ for each sample (**Figure S1D**; **Table S11**). This showed that the miRNA with the largest negative loadings values (positive contribution to AML) were also the most correlated with % cKit+. An unpaired t-test was performed to compare the PC1 components of the CM and control mice at each time point and showed a significant difference between PC1 and % cKit+ cells for timepoints t=1,2,3, and 8 (**Table S2**).

Other dimensionality reduction methods were investigated to construct the AML state-space and were compared to the SVD-derived state-space (**Figure S11**). We observed that the space constructed with diffusion mapping was most similar to the one created with the SVD. This is expected as diffusion mapping uses PCA prior to the application of the diffusion kernel. Nonlinear methods including t-SNE and UMAP did not result in clear separation between the control and CM samples. As a result, the SVD-based state-space was used for the final analysis. The advantage of the SVD-derived state-space is that SVD has no free parameters and maximally preserves the information in the data. All dimensionality reduction algorithms were run with default parameters using the R packages umap v0.2.7.0, Rtsne v0.15, and destiny v3.4.0 for UMAP, t-SNE, and diffusion map respectively.

Pathway analysis of miRNA gene targets
The miRNAs were mapped to experimentally validated gene targets using miRTarBase v8.0 (12). By combining the gene targets with Gene Ontology (GO), KEGG, and WikiPathway databases, pathways and GO terms were converted to their targeting miRNA based on the approach of Godard et al. 2015(13–16). A significant limitation of this approach was that there are currently very few experimentally validated miRNA-gene targets for mice. We therefore limited our investigation of the biological role of our findings in order to avoid over interpretation of the pathway and miRNA-gene target results.

Investigating the AML state-space miRNA loading values
The predicted contribution of each miRNA to AML was tested by identifying miRNA that had been reported to be involved in inv(16) AML previously. The reported role of each miRNA was

noted and then compared to its predicted contribution based on its loading value; large negative loading values have a positive contribution, large positive values have a negative contribution, and loading values near zero have a small contribution. Seven miRNA were found in the literature, and except for those that had small contributions, all predicted contributions miRNA matched the reported role in the literature (**Table S8**). To compare the relationship between the miRNA and AML genes reported in Rockne et al. (*13*), the top 10 miRNA with the highest and lowest contribution to AML were selected from the persistent DE miRNA. The correlation of the expression of these five genes and 20 miRNAs were determined using all CM samples (**Figure 5B**). This showed that the miRNA most correlated with the AML genes were those that had a positive contribution to AML (negative PC1 loading values).

Expression dynamics aligned by critical points

Hierarchical clustering was performed on a correlation matrix consisting of mean centered log2 normalized CPM expression for all CM samples. All miRNA that had zero values in half the CM samples were included in the analysis (894 miRNA). Four expression dynamic groups were identified with the top-level bifurcation of the dendrogram produced by the hierarchical clustering. The expression dynamic plots were created by calculating and plotting the mean expression of all miRNA contained in the group for each CM sample. The mean expression values were then plotted vs PC1.

Angle between mRNA and miRNA principal components

The angle between each of the PCs from mRNA and miRNA (**Figure S9A**) is computed such that for two vectors **a** and **b**, $\theta = \cos^{-1}\left(\frac{\boldsymbol{a}\cdot\boldsymbol{b}}{\|\boldsymbol{a}\|\|\boldsymbol{b}\|}\right)$ where $\|\cdot\|$ is the L-2 norm, or magnitude of the vector.

Supplementary Figures and Tables

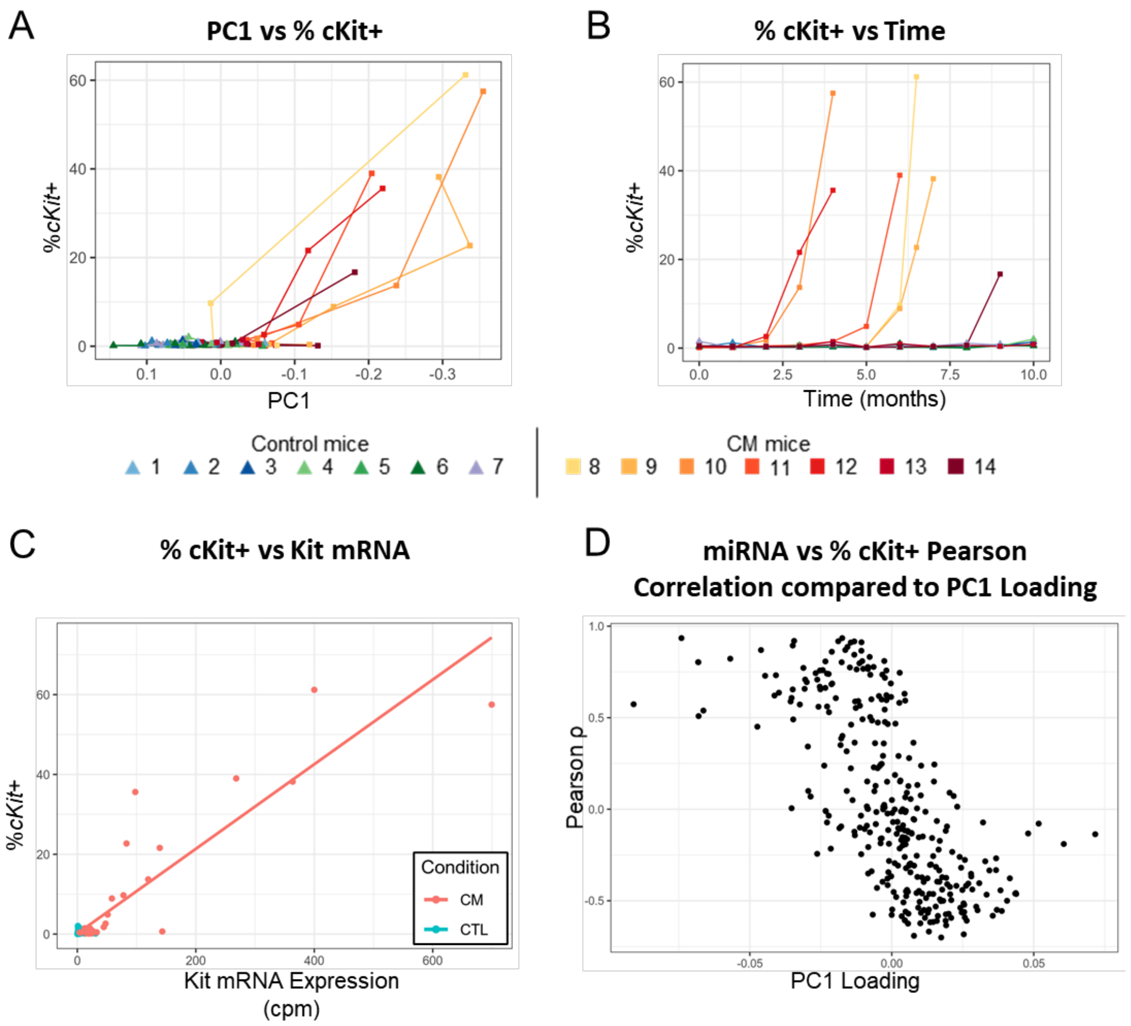

Fig. S1. Kit expression plots.
Kit expression was determined both by flow cytometry using the percent of cells that were cKit positive (% cKit+) and by mRNA expression of *Kit* in each sample. A) % cKit+ is plotted as a function of the AML state-space (PC1 coordinate). B) % cKit+ is plotted as a function of time. In both A) and B), sample trajectories are shown by connecting the time point samples for each mouse. C) %cKit+ and *Kit* mRNA measured as counts per million (cpm) are plotted for each sample to show general agreement between the two measures of Kit. D) For CM mice only, the Pearson correlation coefficient (ρ) was determined between %cKit+ and the expression of each miRNA. Then, the Pearson correlation coefficient was plotted as a function of the PC1 loading value of the miRNA. The result shows that miRNA that are more highly correlated with % cKit+ also tend to have a high contribution to AML (negative PC1 loading value) and vice versa.

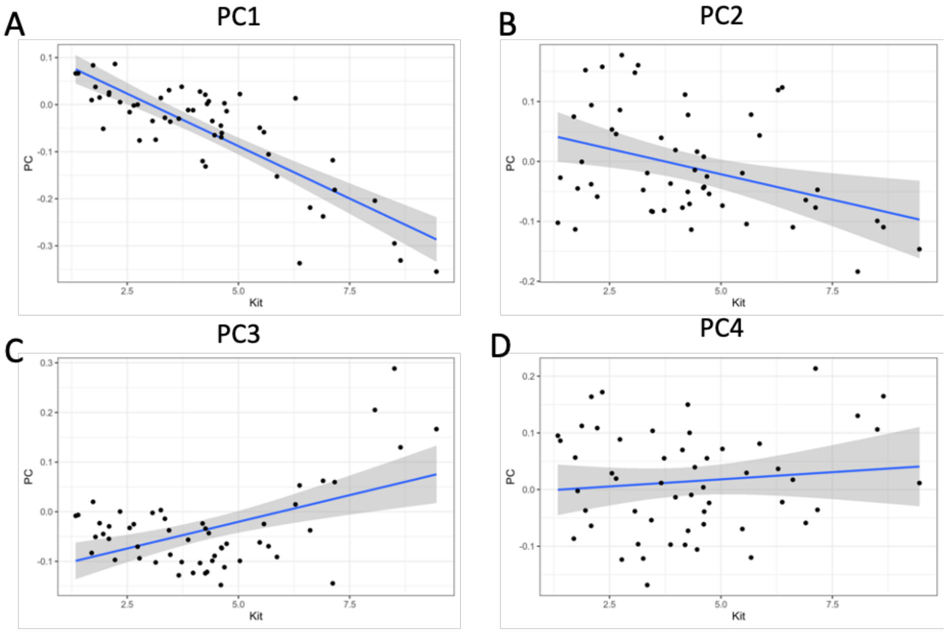

E

PC	R-squared	p-value
1	0.6819	2.75E-14
2	0.1432	0.00521
3	0.2518	0.00013
4	0.0125	0.42493

Fig. S2. Top PCs correlated with Kit Expression.
All principal components (PCs) were tested for correlation with Kit expression. **(A-D)**. The first four PCs, which were above the "elbow" of the singular value plot, are shown. PC1 showed the best correlation (R^2=0.68; $p < 0.001$) of all PCs and was used to define the miRNA AML state-space.

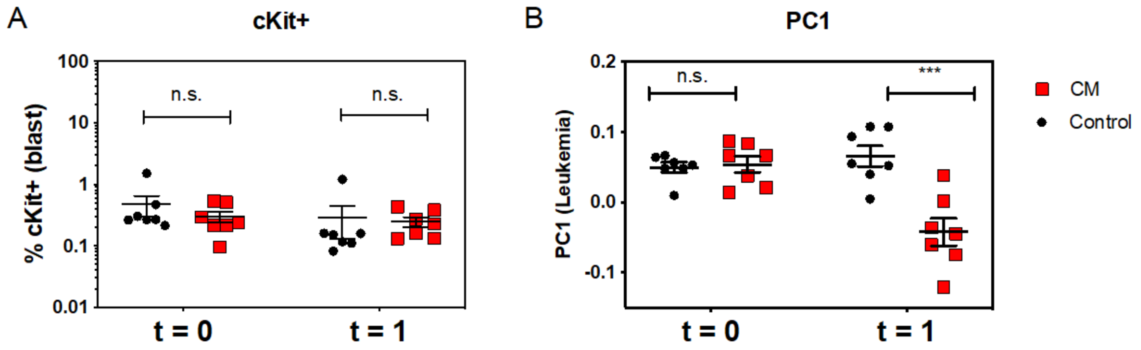

Fig. S3. Kit and PC1 compared between CM vs control mice at early time points.
(A) CM and control sample cKit+ cells are compared at both the pre-induction (t=0) and the first time point after induction of the CM fusion gene (t=1) using flow cytometry as previously described (*13*). In this mouse model, cKit+ cells are an immunophenotypic marker for AML; there were no statistically significant differences between control and CM samples at either time point (unpaired t-test p=0.37 for t=0, p=0.82 for t=1). **(B)** When the same comparison between CM and control is made using the AML state-space component (PC1), the CM samples show a significant decrease in their PC1 component post-induction (unpaired t-test, p<0.01 for t=1). The change in PC1 indicates that the samples have started transitioning toward AML in the state-space. This supports the state-space representation (i.e. the miRNA transcriptome) as an early indicator of state-transition and is able to detect transition toward AML before any cKit+ cells can be detected.

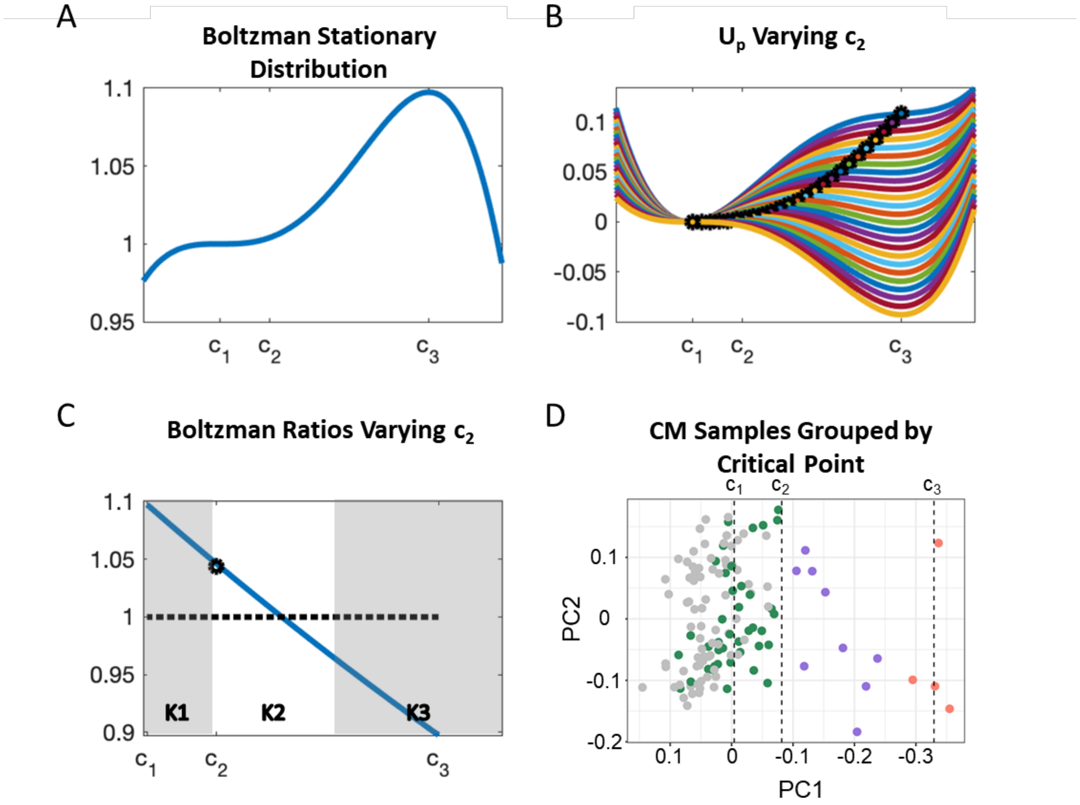

Fig. S4. Estimation of the transition critical point c_2.
(A) The Boltzmann stationary distribution in the state-space is given by $p(x,\infty) = \exp(-\nabla U_p)$. **(B)** The quasi-potential computed for different values of c_2 in the range $c_1 \leq c_2 \leq c_3$, where the location of c_2 is shown as a black circle. As c_2 varies from c_1 to c_3, the shape of the quasi-potential changes, and so too does the predicted stationary distribution. **(C)** To summarize the probability of one state (c_1) or the other (c_3) for various values of c_2, we compute the Boltzmann ratio (B.R.), given by $B.R. = \frac{\exp(-\nabla U_p(c_3))}{\exp(-\nabla U_p(c_1))}$. If $B.R. > 1$, then the state c_3 is more likely, if $B.R. < 1$ then the state c_1 is more likely. We see that the B.R. is maximized for the c_3 state at the boundary of the K1 and K2 clusters. Values of the parameters are given in Table S2. **(D)** State-transition critical points for CM mice are identified in the state-space which characterize the state-transition from health to AML (control samples shown in grey). Using k-means clustering, CM samples were identified as perturbed healthy(c_1 in green), transition (c_2 in purple), or AML ($c_3$in salmon).

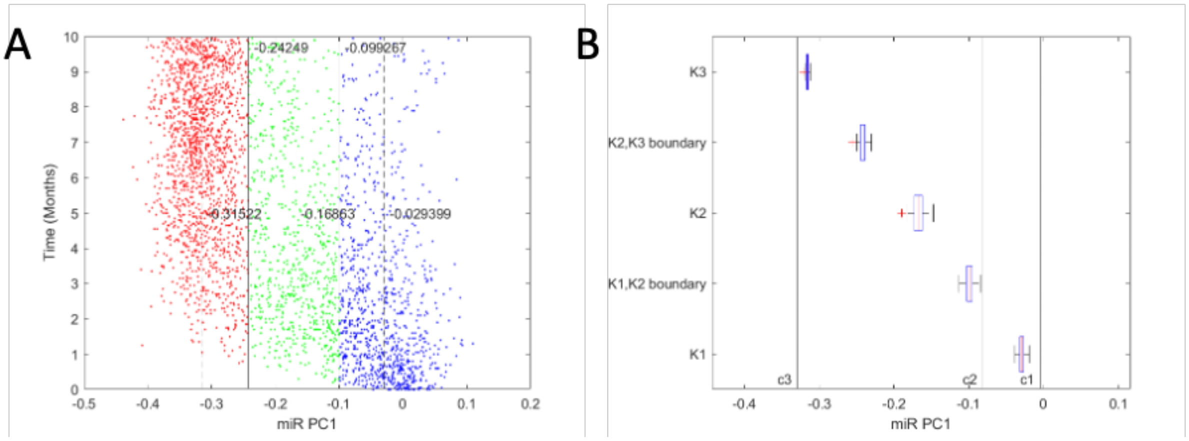

Fig. S5. Simulation study of estimation of transition critical point c_2.
(A) By solving the Langevin equation forward in time to create 1000 virtual state-transition trajectories and randomly sampling 100 points from each trajectory, a virtual state-space is created over time (x-axis PC1, y-axis time). We performed k-means clustering with k=3, (K1 = blue, K2 = green, K3 = red) and plot the cluster means and boundaries between clusters K1-K2 and K2-K3. **(B)** The known critical points used in the simulation c_1, c_2, c_3 are shown as solid lines and the estimators derived from **A)** are shown as box and whisker plots (red line is mean, box is inner quartile, and whiskers are outer quartiles) of the distribution of samples. We see that the mean of K1 is close to c_1, the boundary of K1 and K2 is closest to c_2 and the mean of K3 is closest to c_3, supporting our approach to use these as estimators for the critical points in our model. Actual data is less densely sampled in the state-space and produces slightly different estimators.

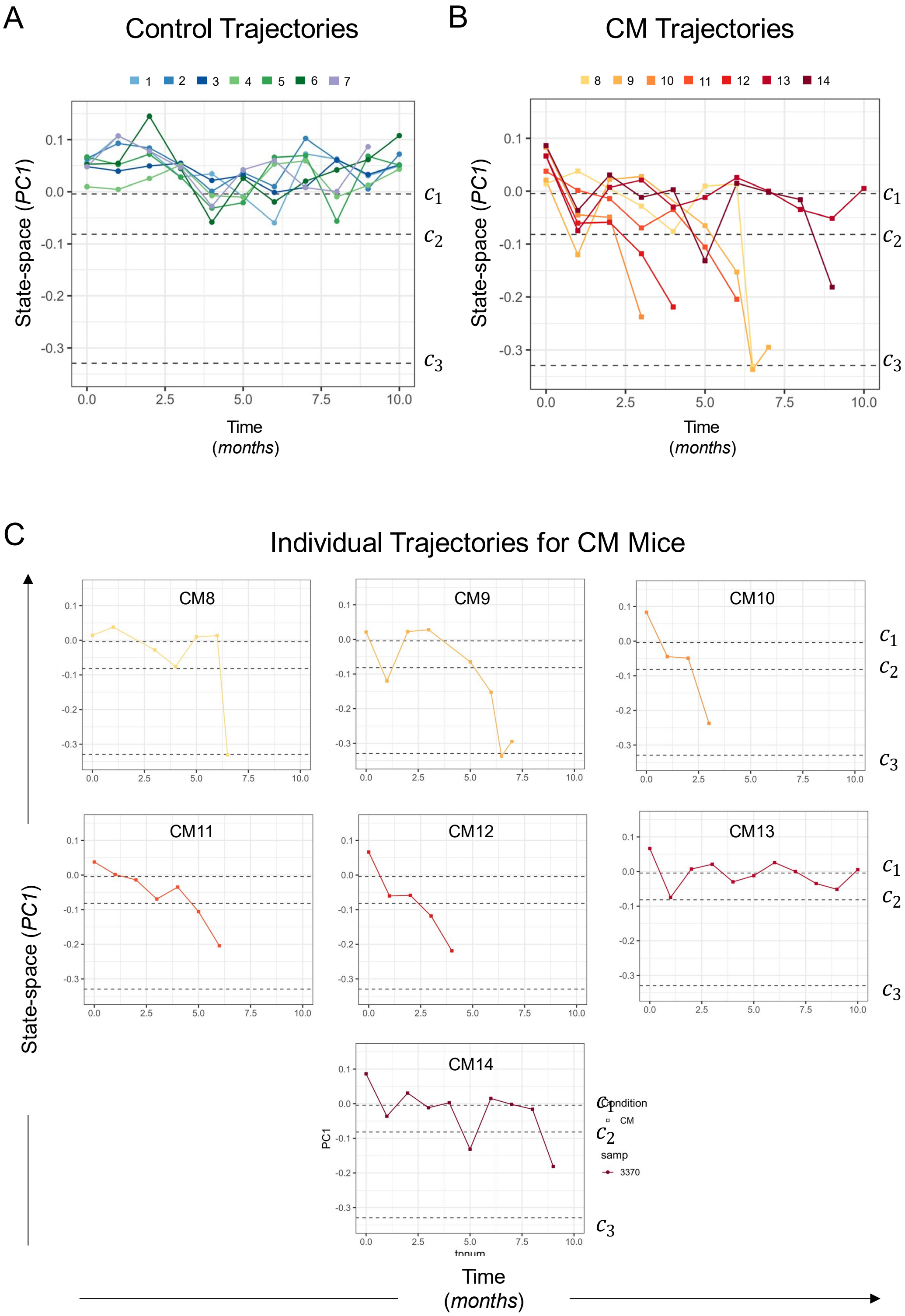

Fig S6. State-space trajectories of CM mice.

The trajectory of each mouse in the AML state-space is shown as function of time, and each mouse is plotted in a different color. Critical points are shown as dashed lines. Except for mouse 13 which never developed AML, all CM mice transition from the state (c_1) toward the AML state (c_3). The control mice remain near the healthy state.

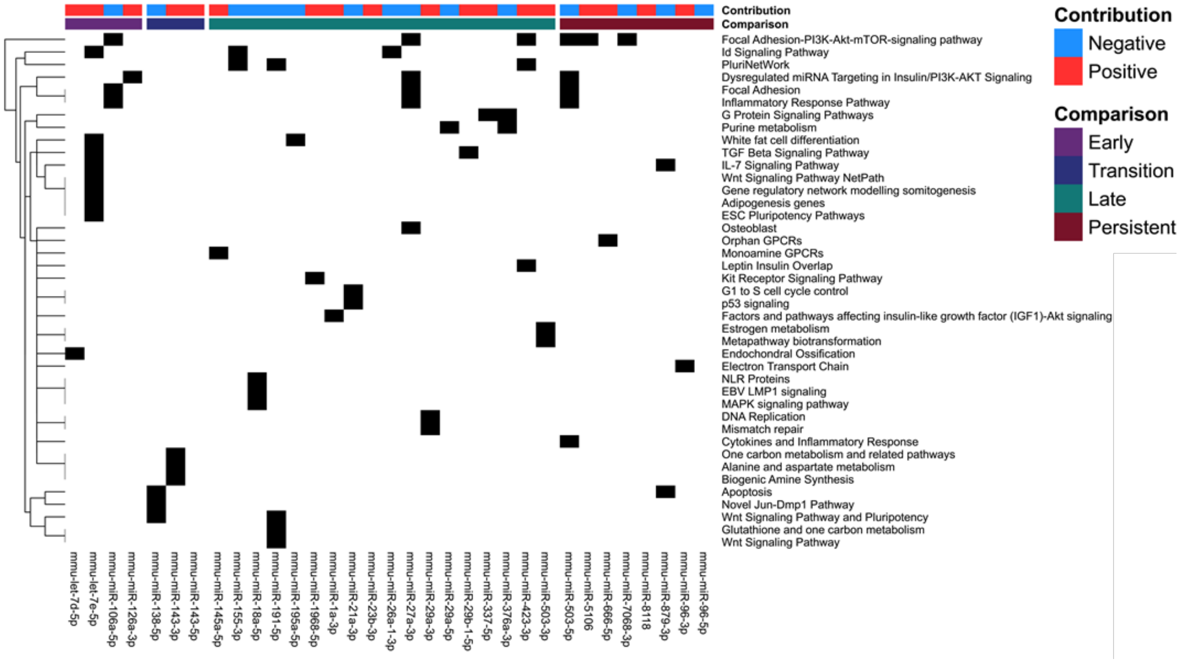

Fig. S7. DE miRNA pathways.

The pathways implicated by DE miRNA for each of the early, transition, late, and persistent events are shown by indicating the miRNA implicated in each pathway (black). Any experimentally validated KEGG or WikiPathway pathway associated with a DE miRNA from the four comparison was reported. The contribution of each miRNA to AML is indicated as either positive (negative loading value) or negative (positive loading value).

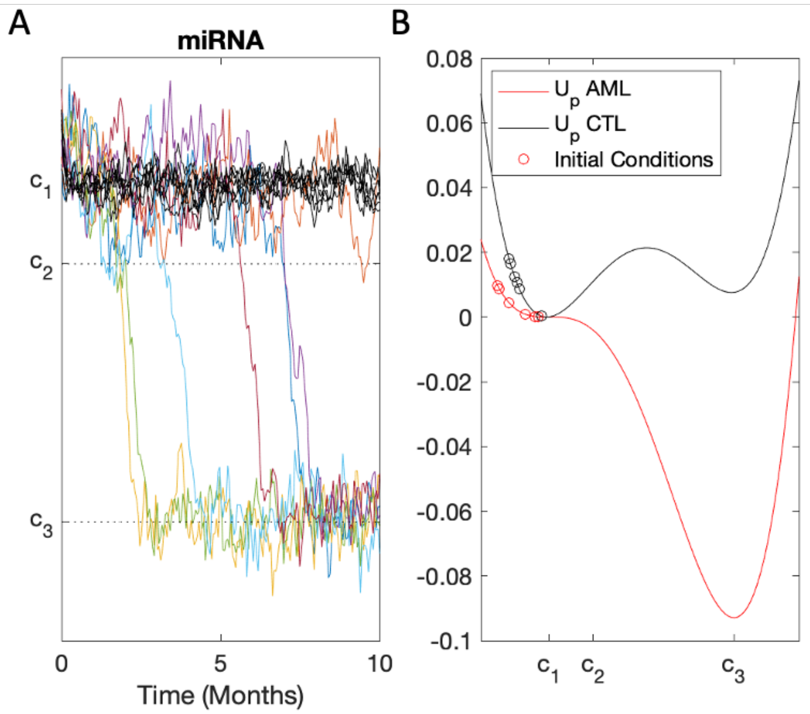

Fig. S8. Simulation of miRNA state-transition dynamics.
(A) By solving the stochastic Langevin equation of motion forward in time $dX_t = -\nabla U_p(X_t)dt + \sqrt{2\beta^{-1}}dB_t$ with parameters and initial conditions (t=1 month post induction) estimated from the validation cohort of CM mice, the model correctly predicts the transition from a state of perturbed hematopoiesis (c_1) to AML (c_3) with different mice (colored lines) manifesting AML at different time points. Controls (black lines) remain in the normal state of hematopoiesis (c_1). **(B)** The quasi-potentials inferred from the data for CM mice undergoing state-transition to AML (red line) and controls (black line). The shape of the AML potential clearly shows a predicted state transition to the lower energy state c_3 as compared to the control potential. Because the equation of motion is stochastic, the prediction of the time to develop AML (**Figure 4B,C**) is given by integrating the corresponding Fokker-Planck probability density function.

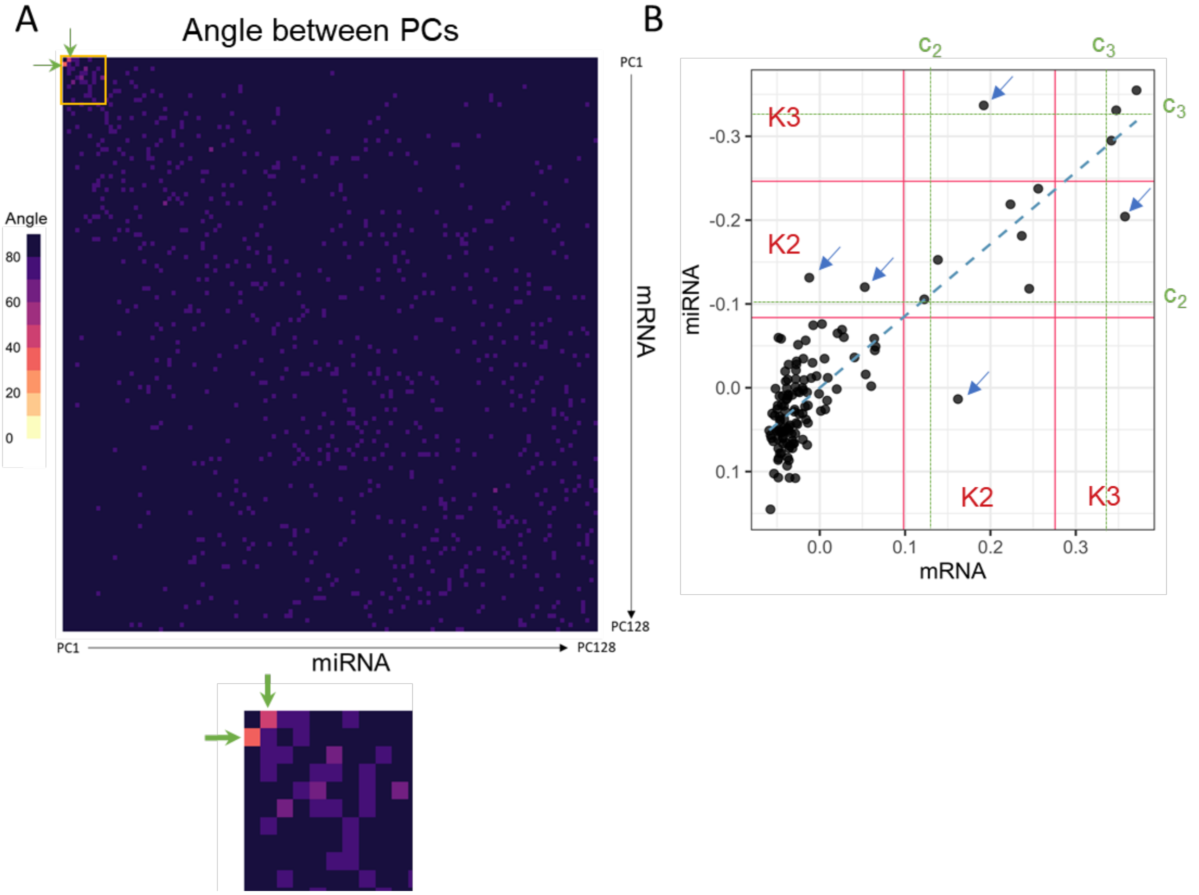

Fig. S9. Comparison of miRNA and mRNA state-spaces.
(A) A heatmap representation of the angle (in degrees) between all non-zero PCs from miRNA (x-axis) and mRNA (y-axis) data. The angle between PCs was determined by taking the inverse cosine of their dot product. An angle close to 90º indicates that the two PCs represent sources of variance within the data that are orthogonal. The PCs with the smallest angle are those corresponding to the AML state-space (miRNA PC1 and mRNA PC2; green arrows, inset). **(B)** State-spaces for miRNA and mRNA plotted against each other annotated with critical points reveals similarities in both the state-transition dynamics and the critical point classification. Only 5 out of 129 samples would be categorized differently depending on which critical points were used, mRNA or miRNA (blue arrows). The linear fit of all samples is shown (blue dashes)

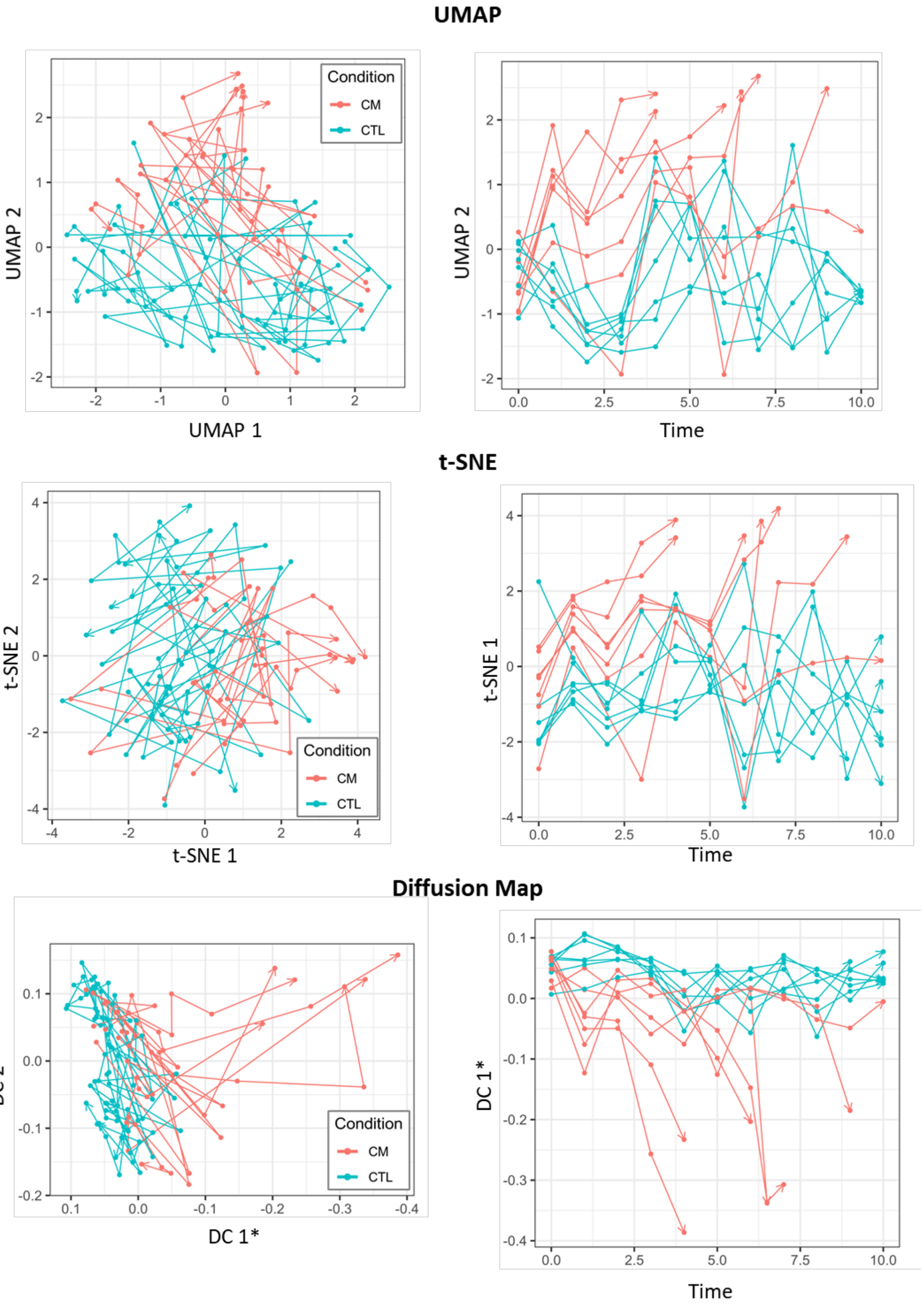

Fig. S10. Alternative dimensionality reduction algorithms.
The miRNA state-space is constructed using different dimensionality reduction algorithms: UMAP, t-SNE, and Diffusion Map to compare with the state-space constructed with the singular value decomposition. For each algorithm, plots were made showing both the first two components and the component that best separates the AML samples is plotted versus time. The SVD was selected to create the state-space and for analysis in the main text because it is a linear

method and requires no free parameters, in contrast to the free parameters; perplexity (UMAP, t-SNE) and the variance of the Gaussian kernel (Diffusion Map).

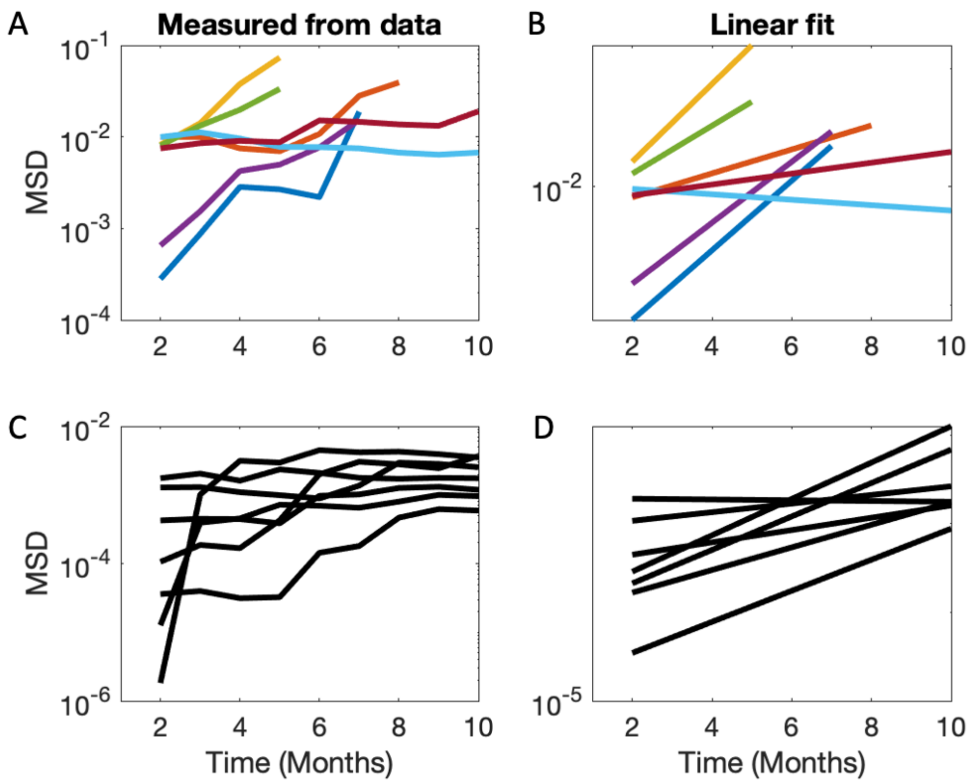

Fig. S11. Mean squared displacement analysis.
Mean squared displacement (MSD) analysis of miRNA trajectories in the AML state-space. **(A)** MSD computed from PC1 trajectories for n=7 CM mice with log-scale y-axis. Each mouse trajectory is a different color, coordinated with the panel on the right. **(B)** Linear fit of the MSDs for each CM mouse. The mean of the slopes of the linear fits is used as an estimator of the diffusion coefficient β in both the Langevin equation of motion and Fokker-Planck probability density model. **(C-D)** Same analysis for control mice. The slopes of the MSD curves and linear fits are smaller for the control mice as compared to the CM mice. The flat MSD curves and reduced slopes for the control mice suggesting confined diffusion, as compared to the CM mice. Diffusion parameters are shown in Table S2.

Table S1. Kit expression correlation table. (separate file)
Kit mRNA expression was correlated with each principal component produced by performing singular value decomposition on miRNA expression. Correlation R^2 and p-values were reported.

Table S2. PC1 CM vs control t-tests.
Multiple unpaired t-tests with Welch correction to test the null hypothesis that the difference in PC1 between control and CM samples is zero with false discovery rate 0.01 and significance level 0.05. Differences are statistically significant at timepoints t=1,2,3,5, and 8. Standard error (SE).

	**Significant?**	**P-value**	**Mean of CTRL**	**Mean of CM**	**Difference**	**SE of difference**
t=0	No	0.752933	0.04943	0.05369	-0.004263	0.01318
**t=1**	**Yes**	0.000960	0.06554	-0.04231	0.1079	0.02427
**t=2**	**Yes**	0.001608	0.07611	-0.01019	0.08631	0.02058
**t=3**	**Yes**	0.026812	0.04367	-0.05936	0.1030	0.03563
t=4	No	0.115786	-0.009718	-0.1186	0.1088	0.05826
**t=5**	**Yes**	0.046836	0.01371	-0.06079	0.07450	0.02856
t=6	No	0.203053	0.01556	-0.06044	0.07600	0.05200
t=7	No	0.269865	0.04867	-0.09886	0.1475	0.09896
**t=8**	**Yes**	0.046496	0.02301	-0.02530	0.04831	0.01978
t=9	No	0.238416	0.04269	-0.1163	0.1589	0.06594

Table S3. State-space coordinates. (separate file)
Coordinates of each sample in the AML state-space. The 2D AML state-space was constructed by plotting PC1 vs PC2 for each sample.

Table S4. **Critical points and simulation parameters**.
For controls, c_2 was taken to be the midpoint between c_1 and c_3. Parameters were estimated as described in main text and supplemental methods.

**Parameter**	**Meaning**	**Value**
$\boldsymbol{c_1}$	Normal hematopoiesis	-0.0042
$\boldsymbol{c_2}$	Transition state	-0.0816
$\boldsymbol{c_3}$	AML	-0.3294
$\boldsymbol{\alpha}$	Scaling factor of quasi-potential	100
$\boldsymbol{\beta}$	Diffusion coefficient	285.7143
$\boldsymbol{\beta_H}$	Diffusion coefficient for controls	1.4286e+03

Table S5. DE miRNA lists. (separate file)
The differentially expressed (DE) miRNA that define the early, transition, late, persistent event in AML were reported.

Table S6. miRNA in each expression dynamics group. (separate file)
The table lists the miRNA contained within each of the four dynamic expression groups.

Table S7. Pathway summary key. (separate file)
The pathways implicated for each of the expression dynamic groups were grouped to summarize their function. This table includes their grouping and summarized function.

Table S8. Inv(16) AML miRNA.
miRNA previously reported as being associated with inv(16) AML were used to compare their reported expression in AML to their predicted contribution to AML based on their PC1 loading value.

miRNA	PC1 Loading	AML Contribution	Direction in AML	Concurrent?	Reference
miR-126a-3p	-0.0681	Positive	Up	Yes	Li et al. 2008 (*31*); Zhang et al. 2021 (*9*)
miR-126a-5p	-0.0682	Positive	Up	Yes	Li et al. 2008 (*31*)
miR-92-3p	-0.0019	Small	Down	NA	Li et al. 2008 (*31*)
miR-99a-5p	0.0048	Small	Up (vs other AMLs)	NA	Dixon-McIver et al. 2008 (*32*)
miR-100-5p	-0.0132	Positive	Up (vs other AMLs)	Yes	Dixon-McIver et al. 2008 (*32*)
miR-224-5p	-0.0088	Small	Up (vs other AMLs)	NA	Dixon-McIver et al. 2008 (*32*)
miR-142-3p	0.0233	Negative	Down	Yes	Cammarata et al. 2010 (*33*)
miR-99-3p	0.0146	Negative	Down	Yes	Cammarata et al. 2010 (*33*)

Table S9. PC1 loading values for each miRNA. (separate file)
For each miRNA, the table records the associated PC1 loading values.

Table S10. cKit positive values. (separate file)
Flow cytometry was used to determine the percent of cells that were *cKit* positive (%*cKit*+) for each sample.

Table S11. miRNA vs Kit correlation table. (separate file)
%*cKit*+ values were correlated with the expression of each miRNA. R^2 and p-values are reported.

Table S12. Sample IDs. (separate file)
The table provides a key to the sample names and sample identifiers used in the manuscript figures.

REFERENCES AND NOTES

1. A. Noone, N. Howlader, M. Krapcho, D. Miller, A. Brest, M. Yu, J. Ruhl, Z. Tatalovich, A. Mariotto, D. Lewis, H. Chen, E. Feuer, K. Cronin, *SEER Cancer Statistics Review* (National Cancer Institute, 2020).

2. P. Ao, D. Galas, L. Hood, X. Zhu, Cancer as robust intrinsic state of endogenous molecular-cellular network shaped by evolution. *Med. Hypotheses* **70**, 678–684 (2008).

3. S. Huang, I. Ernberg, S. Kauffman, Cancer attractors: A systems view of tumors from a gene network dynamics and developmental perspective. *Semin. Cell Dev. Biol.* **20**, 869–876 (2009).

4. Q. Cai, R. Jeannet, W. K. Hua, G. J. Cook, B. Zhang, J. Qi, H. Liu, L. Li, C. C. Chen, G. Marcucci, Y. H. Kuo, CBFβ-SMMHC creates aberrant megakaryocyte-erythroid progenitors prone to leukemia initiation in mice. *Blood* **128**, 1503–1515 (2016).

5. Y. H. Kuo, S. F. Landrette, S. A. Heilman, P. N. Perrat, L. Garrett, P. P. Liu, M. M. Le Beau, S. C. Kogan, L. H. Castilla, Cbf beta-SMMHC induces distinct abnormal myeloid progenitors able to develop acute myeloid leukemia. *Cancer Cell* **9**, 57–68 (2006).

6. W. G. Bardsley, The reduction in degree of allosteric and other complex rate equations using Sylvester’s dialytic method of elimination. *J. Theor. Biol.* **67**, 121–139 (1977).

7. P. Liu, S. A. Tarlé, A. Hajra, D. F. Claxton, P. Marlton, M. Freedman, M. J. Siciliano, F. S. Collins, Fusion between transcription factor CBF beta/PEBP2 beta and a myosin heavy chain in acute myeloid leukemia. *Science* **261**, 1041–1044 (1993).

8. M. I. Love, W. Huber, S. Anders, Moderated estimation of fold change and dispersion for RNA-seq data with DESeq2. *Genome Biol.* **15**, 550 (2014).

9. L. Zhang, L. X. T. Nguyen, Y.-C. Chen, D. Wu, G. J. Cook, D. H. Hoang, C. J. Brewer, X. He, H. Dong, S. Li, M. Li, D. Zhao, J. Qi, W.-K. Hua, Q. Cai, E. Carnahan, W. Chen, X. Wu, P. Swiderski, R. C. Rockne, M. Kortylewski, L. Li, B. Zhang, G. Marcucci, Y.-H. Kuo, Targeting miR-126 in

inv(16) acute myeloid leukemia inhibits leukemia development and leukemia stem cell maintenance. *Nat. Commun.* **12**, 6154 (2021).

10. C. Z. Chen, L. Li, H. F. Lodish, D. P. Bartel, MicroRNAs modulate hematopoietic lineage differentiation. *Science* **303**, 83–86 (2004).

11. R. Su, H. S. Lin, X. H. Zhang, X. L. Yin, H. M. Ning, B. Liu, P. F. Zhai, J. N. Gong, C. Shen, L. Song, J. Chen, F. Wang, H. L. Zhao, Y. N. Ma, J. Yu, J. W. Zhang, MiR-181 family: Regulators of myeloid differentiation and acute myeloid leukemia as well as potential therapeutic targets. *Oncogene* **34**, 3226–3239 (2015).

12. T. Kehl, F. Kern, C. Backes, T. Fehlmann, D. Stöckel, E. Meese, H. P. Lenhof, A. Keller, MiRPathDB 2.0: A novel release of the miRNA pathway dictionary database. *Nucleic Acids Res.* **48**, D142–D147 (2020).

13. R. C. Rockne, S. Branciamore, J. Qi, D. E. Frankhouser, D. O'Meally, W. K. Hua, G. Cook, E. Carnahan, L. Zhang, A. Marom, H. Wu, D. Maestrini, X. Wu, Y. C. Yuan, Z. Liu, L. D. Wang, S. Forman, N. Carlesso, Y. H. Kuo, G. Marcucci, State-transition analysis of time-sequential gene expression identifies critical points that predict development of acute myeloid leukemia. *Cancer Res.* **80**, 3157–3169 (2020).

14. T. Yu, P. Ma, D. Wu, Y. Shu, W. Gao, Functions and mechanisms of microRNA-31 in human cancers. *Biomed. Pharmacother.* **108**, 1162–1169 (2018).

15. E. M. Laurila, A. Kallioniemi, The diverse role of miR-31 in regulating cancer associated phenotypes. *Genes Chromosom. Cancer* **52**, 1103–1113 (2013).

16. Z. Huang, Y. Xu, M. Wan, X. Zeng, J. Wu, miR-340: A multifunctional role in human malignant diseases. *Int. J. Biol. Sci.* **17**, 236 (2021).

17. Q. Wang, T. Feng, J. Xu, M. H. Miao, X. Q. Ji, H. Zhu, X. J. Shao, Low expression of microRNA-340 confers adverse clinical outcome in patients with acute myeloid leukemia. *J. Cell. Physiol.* **234**, 4200–4205 (2019).

18. S. Kauffman, A proposal for using the ensemble approach to understand genetic regulatory networks. *J. Theor. Biol.* **230**, 581–590 (2004).

19. S. Huang, G. Eichler, Y. Bar-Yam, D. E. Ingber, Cell fates as high-dimensional attractor states of a complex gene regulatory network. *Phys. Rev. Lett.* **94**, 128701 (2005).

20. J. C. Mar, J. Quackenbush, Decomposition of gene expression state space trajectories. *PLoS Comput. Biol.* **5**, e1000626 (2009).

21. O. Alter, P. O. Brown, D. Botstein, Singular value decomposition for genome-Wide expression data processing and modeling. *Proc. Natl. Acad. Sci. U.S.A.* **97**, 10101–10106 (2000).

22. P. Sankaranarayanan, T. E. Schomay, K. A. Aiello, O. Alter, Tensor GSVD of patient- and platform-matched tumor and normal DNA copy-number profiles uncovers chromosome arm-wide patterns of tumor-exclusive platform-consistent alterations encoding for cell transformation and predicting ovarian cancer survival. *PLOS ONE* **10**, e0121396 (2015).

23. M. W. Bradley, K. A. Aiello, S. P. Ponnapalli, H. A. Hanson, O. Alter, GSVD- and tensor GSVD-uncovered patterns of DNA copy-number alterations predict adenocarcinomas survival in general and in response to platinum. *APL Bioeng.* **3**, 036104 (2019).

24. S. N. Naccache, S. Federman, N. Veeraraghavan, M. Zaharia, D. Lee, E. Samayoa, J. Bouquet, A. L. Greninger, K. C. Luk, B. Enge, D. A. Wadford, S. L. Messenger, G. L. Genrich, K. Pellegrino, G. Grard, E. Leroy, B. S. Schneider, J. N. Fair, M. A. Martínez, P. Isa, J. A. Crump, J. L. DeRisi, T. Sittler, J. Hackett, S. Miller, C. Y. Chiu, A cloud-compatible bioinformatics pipeline for ultrarapid pathogen identification from next-generation sequencing of clinical samples. *Genome Res.* **24**, 1180–1192 (2014).

25. T. T. Bui, K. Selvarajoo, Attractor concepts to evaluate the transcriptome-wide dynamics guiding anaerobic to aerobic state transition in Escherichia coli. *Sci. Rep.* **10**, 5878 (2020).

26. R. Yuan, P. Ao, G. Wang, H. Su, Coarse-grained endogenous network predicts main dynamical sequences in myeloid differentiation and trans-differentiation. *Sci. Sin. Vitae* **47**, 616–628 (2017).

27. K. A. Lawson, C. M. Sousa, X. Zhang, E. Kim, R. Akthar, J. J. Caumanns, Y. Yao, N. Mikolajewicz, C. Ross, K. R. Brown, A. A. Zid, Z. P. Fan, S. Hui, J. A. Krall, D. M. Simons, C. J. Slater, V. De Jesus, L. Tang, R. Singh, J. E. Goldford, S. Martin, Q. Huang, E. A. Francis, A. Habsid, R. Climie, D. Tieu, J. Wei, R. Li, A. H. Y. Tong, M. Aregger, K. S. Chan, H. Han, X. Wang, P. Mero, J. H. Brumell, A. Finelli, L. Ailles, G. Bader, G. A. Smolen, G. A. Kingsbury, T. Hart, C. Kung, J. Moffat, Functional genomic landscape of cancer-intrinsic evasion of killing by T cells. *Nature* **586**, 120–126 (2020).

28. M. Mojtahedi, A. Skupin, J. Zhou, I. G. Castaño, R. Y. Y. Leong-Quong, H. Chang, K. Trachana, A. Giuliani, S. Huang, Cell fate decision as high-dimensional critical state transition. *PLoS Biol.* **14**, e2000640 (2016).

29. P. Ao, Global view of bionetwork dynamics: Adaptive landscape. *J. Genet. Genomics* **36**, 63–73 (2009).

30. H. Venkatachalapathy, S. M. Azarin, C. A. Sarkar, Trajectory-based energy landscapes of gene regulatory networks. *Biophys. J.* **120**, 687–698 (2021).

31. Z. Li, J. Lu, M. Sun, S. Mi, H. Zhang, R. T. Lou, P. Chen, Y. Wang, M. Yan, Z. Qian, M. B. Neilly, J. Jin, Y. Zhang, S. K. Bohlander, D.-E. Zhang, R. A. Larson, M. M. Le Beau, M. Thirman, T. R. Golub, J. D. Rowley, J. Chen, Distinct microRNA expression profiles in acute myeloid leukemia with common translocations. *Proc. Natl. Acad. Sci. U.S.A.* **105**, 15535–15540 (2008).

32. A. Dixon-McIver, P. East, C. A. Mein, J.-B. Cazier, G. Molloy, T. Chaplin, T. A. Lister, B. D. Young, S. Debernardi, Distinctive patterns of MicroRNA expression associated with karyotype in acute myeloid leukaemia. *PLOS ONE* **3**, e2141 (2008).

33. G. Cammarata, L. Augugliaro, D. Salemi, C. Agueli, M. La Rosa, L. Dagnino, G. Civiletto, F. Messana, A. Marfia, M. G. Bica, L. Cascio, P. M. Floridia, A. M. Mineo, M. Russo, F. Fabbiano, A. Santoro, Differential expression of specific microRNA and their targets in acute myeloid leukemia. *Am. J. Hematol.* **85**, 331–339 (2010).

34. P. A. Ewels, A. Peltzer, S. Fillinger, J. Alneberg, H. Patel, A. Wilm, M. U. Garcia, P. Di Tommaso, S. Nahnsen, nf-core: Community curated bioinformatics pipelines. *bioRxiv*, 610741 (2019).

35. B. Langmead, S. L. Salzberg, Fast gapped-read alignment with Bowtie 2. *Nat. Methods* **9**, 357–359 (2012).

36. A. Kozomara, M. Birgaoanu, S. Griffiths-Jones, MiRBase: From microRNA sequences to function. *Nucleic Acids Res.* **47**, D155–D162 (2019).

37. H. Li, B. Handsaker, A. Wysoker, T. Fennell, J. Ruan, N. Homer, G. Marth, G. Abecasis, R. Durbin, The sequence alignment/map format and SAMtools. *Bioinformatics* **25**, 2078–2079 (2009).

38. M. D. Robinson, D. J. McCarthy, G. K. Smyth, edgeR: A Bioconductor package for differential expression analysis of digital gene expression data. *Bioinformatics* **26**, 139–140 (2010).

39. J. T. Leek, W. E. Johnson, H. S. Parker, A. E. Jaffe, J. D. Storey, The SVA package for removing batch effects and other unwanted variation in high-throughput experiments. *Bioinformatics* **28**, 882–883 (2012).

40. J. Wagner, M. A. Rapsomaniki, S. Chevrier, T. Anzeneder, C. Langwieder, A. Dykgers, M. Rees, A. Ramaswamy, S. Muenst, S. D. Soysal, A. Jacobs, J. Windhager, K. Silina, M. van den Broek, K. J. Dedes, M. Rodríguez Martínez, W. P. Weber, B. Bodenmiller, A single-cell atlas of the tumor and immune ecosystem of human breast cancer. *Cell* **177**, 1330–1345.e18 (2019).

41. L. Pantano, miRTOP.github.io: Basic structure for home page (2016);10.5281/ZENODO.45385.

42. M. Martin, Cutadapt removes adapter sequences from high-throughput sequencing reads. *EMBnet J.* **17**, 10 (2011).

43. B. Langmead, C. Trapnell, M. Pop, S. L. Salzberg, Ultrafast and memory-efficient alignment of short DNA sequences to the human genome. *Genome Biol.* **10**, R25 (2009).
